# Molecular basis of the glycosomal targeting of PEX11 and its mislocalization to mitochondrion in trypanosomes

**DOI:** 10.3389/fcell.2023.1213761

**Published:** 2023-08-17

**Authors:** Chethan K. Krishna, Nadine Schmidt, Bettina G. Tippler, Wolfgang Schliebs, Martin Jung, Konstanze F. Winklhofer, Ralf Erdmann, Vishal C. Kalel

**Affiliations:** ^1^ Department of Systems Biochemistry, Institute for Biochemistry and Pathobiochemistry, Faculty of Medicine, Ruhr University Bochum, Bochum, Germany; ^2^ Department of Medical Biochemistry and Molecular Biology, Saarland University, Homburg, Germany; ^3^ Department Molecular Cell Biology, Institute of Biochemistry and Pathobiochemistry, Faculty of Medicine, Ruhr University Bochum, Bochum, Germany

**Keywords:** peroxisome, glycosome, peroxin, PEX19, PMP, mPTS, MTS

## Abstract

PEX19 binding sites are essential parts of the targeting signals of peroxisomal membrane proteins (mPTS). In this study, we characterized PEX19 binding sites of PEX11, the most abundant peroxisomal and glycosomal membrane protein from *Trypanosoma brucei* and *Saccharomyces cerevisiae*. *Tb*PEX11 contains two PEX19 binding sites, one close to the N-terminus (BS1) and a second in proximity to the first transmembrane domain (BS2). The N-terminal BS1 is highly conserved across different organisms and is required for maintenance of the steady-state concentration and efficient targeting to peroxisomes and glycosomes in both baker’s yeast and *Trypanosoma brucei*. The second PEX19 binding site in *Tb*PEX11 is essential for its glycosomal localization. Deletion or mutations of the PEX19 binding sites in *Tb*PEX11 or *Sc*PEX11 results in mislocalization of the proteins to mitochondria. Bioinformatic analysis indicates that the N-terminal region of *Tb*PEX11 contains an amphiphilic helix and several putative TOM20 recognition motifs. We show that the extreme N-terminal region of *Tb*PEX11 contains a cryptic N-terminal signal that directs PEX11 to the mitochondrion if its glycosomal transport is blocked.

## 1 Introduction

Peroxisomes are single membrane bound organelles performing a wide range of functions (Rhodin, 1954; De Duve and Baudhuin, 1966). Glyoxysomes in plants, Woronin bodies in fungi, and glycosomes in trypanosomatid parasites are specialized forms of peroxisomes (Reichle and Alexander, 1965; Breidenbach and Beevers, 1967; Opperdoes and Borst, 1977). Peroxisomes can multiply by growth and division, or they can form *de novo* from pre-peroxisomal vesicles that are supposed to bud from the endoplasmic reticulum (Hoepfner et al., 2005; Motley and Hettema, 2007). Peroxisomes import matrix as well as membrane proteins post-translationally (Goldman Blobel, 1978; Lazarow and Fujiki, 1985; Sacksteder et al., 2000; Jones et al., 2004). The import depends on a machinery of Peroxins (PEX proteins) and requires the presence of peroxisomal targeting signals in the cargo proteins (Gould et al., 1989; Swinkels et al., 1991; Faber et al., 1995). Biogenesis of peroxisomes requires two distinct machineries for protein targeting: The first is responsible for the formation of the peroxisomal membrane by the targeting and insertion of peroxisomal membrane proteins (PMPs), and the second machinery is responsible for the import of peroxisomal matrix proteins (reviewed in (Agrawal and Subramani, 2016)). The trafficking of proteins destined for the peroxisome matrix has been well studied. A striking feature is that peroxisomes can import folded, even oligomeric proteins (McNew andGoodman, 1994; Leon et al., 2006). Peroxisomal matrix proteins contain type 1 or type 2 peroxisomal targeting signals (PTS1/PTS2) at the extreme C-terminus or close to the N-terminus, respectively (Gould et al., 1989; Swinkels et al., 1991; Faber et al., 1995). Some proteins contain internal targeting signals (Galland et al., 2010) and some are transported by piggy-backing onto a PTS-containing protein (Islinger et al., 2009). The import of peroxisomal matrix proteins depends on cycling receptors that recognize peroxisomal proteins via their PTS in the cytosol and target them to a docking complex at the peroxisomal membrane. Import takes place through a transient pore or hydrogel-filled pore in an unknown fashion (Erdmann and Schliebs, 2005; Meinecke et al., 2016; Gao et al., 2022). The cargo-unloaded receptors are mono-ubiquitinated and released to the cytosol for another round of import in an ATP-dependent manner by the peroxisomal exportomer (Platta et al., 2004; Platta et al., 2005). The machinery that is responsible for the topogenesis of membrane proteins is distinct from the import machinery for matrix proteins (Reviewed in (Hasan et al., 2013; Mayerhofer, 2016)). Only three peroxins with a direct role in PMP targeting have been identified, namely, PEX3 (Hettema et al., 2000), PEX16 in mammals (South and Gould, 1999; Sacksteder et al., 2000), and PEX19 (Sacksteder et al., 2000) (Also reviewed in (Kalel and Erdmann, 2018)). In cells lacking any of these proteins, PMPs are either degraded or mistargeted to other subcellular compartments such as mitochondria, endoplasmic reticulum (ER), and membranes of unknown origin (Ghaedi et al., 2000; Hettema et al., 2000; Sacksteder et al., 2000). PMPs contain multiple binding sites (BSs) for the cytosolic receptor and chaperone PEX19 (Jones et al., 2004). These binding sites are essential for targeting of the PMPs to the peroxisomal membrane, as they can function as mPTS i.e., membrane peroxisome targeting signal (Halbach et al., 2005). The mPTS often comprises part of the transmembrane domains and a short adjacent sequence, which contains either a cluster of basic residues or a mixture of basic and hydrophobic amino acids (Marshall et al., 1996) (Reviewed in (Baerends et al., 2000; Murphy et al., 2003; Van Ael and Fransen, 2006)). Rottensteiner et al., developed PEX19 binding site prediction methodology using peptide arrays (Rottensteiner et al., 2004). Unlike PTS1 and PTS2 signals, which can be predicted more reliably (Kamoshita et al., 2022; Kunze, 2023), PEX19 BSs are comparatively degenerate and can be present multiple times in a PMP. Therefore, an efficient PEX19BS predictor is still needed. Nonetheless, PEX19 binding sites (BSs) have been identified in various yeast, human and parasite PMPs, which shows evolutionary conservation across eukaryotes (Rottensteiner et al., 2004; Saveria et al., 2007). In most eukaryotes, PEX19 harbors a farnesylation motif (CaaX box), and farnesylation has been shown to increase the binding efficiency of PMPs (Rucktaschel et al., 2009). However, trypanosomatid parasite PEX19 proteins lack such a CaaX motif (Banerjee et al., 2005).

PEX11 is an integral peroxisomal membrane protein with at least two predicted alpha-helical transmembrane domains and both termini facing the cytosol (Abe et al., 1998b; Lorenz et al., 1998; Anton et al., 2000; Bonekamp et al., 2013). In the yeast *Saccharomyces cerevisiae*, Pex11p, Pex25p, and Pex27p are the three members of the PEX11 protein family (Erdmann and Blobel, 1995; Rottensteiner et al., 2003; Tam et al., 2003). Similarly, mammals also encode three PEX11-family proteins namely, PEX11α, PEX11β, and PEX11γ (Li and Gould, 2002; Koch et al., 2010). In plants, there are five PEX11 homologs, for e. g., in *Arabidopsis thaliana At*PEX11a, -b, -c, -d, and -e (Lingard and Trelease, 2006). PEX11 family proteins are involved in the proliferation of peroxisome in yeasts, plants, and mammals (Erdmann and Blobel, 1995; Abe and Fujiki, 1998a; Schrader et al., 1998; Orth et al., 2007; Koch et al., 2010). Deletion of PEX11 in yeast has an effect on the β-oxidation of fatty acids, which can be due to defects in the transport of metabolites across the peroxisomal membrane (Sulter et al., 1993). PEX11β is widely expressed in mammalian tissues and it has a well-recognized function in the initial phase of peroxisomal fission when it remodels and elongates peroxisomal membranes (Delille et al., 2010; Yoshida et al., 2015; Schrader et al., 2016). The functions of PEX11α and PEX11γ are less clear (Schrader et al., 2016). Of the PEX11 proteins in mammals, only PEX11β deficiency was associated with the pathology of peroxisome biogenesis disorders (PBDs) (Li and Gould, 2002; Thoms and Gartner, 2012; Schrader et al., 2016).

In trypanosomes, three PEX11 family proteins are known, namely, PEX11, GIM5A and GIM5B (Lorenz et al., 1998; Maier et al., 2001; Voncken et al., 2003). Like in mammals, yeast, and plants, both N- and C-termini of *Tb*PEX11 are exposed to the cytosol (Lorenz et al., 1998). Overexpression of *Tb*PEX11 induces growth inhibition and transforms the globular glycosomes into long tubule clusters that occupy a large portion of the cytoplasm (Lorenz et al., 1998). Accordingly, *Tb*PEX11 appears to play a role in the proliferation of glycosomes in trypanosomes like its homologs in yeast and mammalian cells (van Roermund et al., 2000) (Reviewed in (Moyersoen et al., 2004)). PEX11 and both GIM5 proteins are essential for the survival of parasites (Lorenz et al., 1998; Voncken et al., 2003). At primary sequence level, PEX11 family proteins contain several conserved helices particularly in the N-terminal region (Lorenz et al., 1998; Opalinski et al., 2018). PEX19 binding sites have been identified in various glycosomal membrane proteins (Saveria et al., 2007), but not in *Tb*PEX11. Therefore, in this study, we characterized PEX19 binding sites of PEX11 from *Trypanosoma brucei* and *Saccharomyces cerevisiae*. *Tb*PEX11 contains two PEX19 binding sites, the N-terminal PEX19 binding site (BS1) in PEX11 is highly conserved across different organisms and is required for maintenance of the steady-state concentration as well as efficient targeting to peroxisomes and glycosomes in both baker’s yeast and *T. brucei*. Deletion or mutations of the PEX19 binding site in *Tb*PEX11 (second PEX19 BS, i.e., BS2) or *Sc*PEX11 (single PEX19 BS) results in a mislocalization of the proteins to mitochondria. Trypanosomes contain multiple small glycosomes, but harbor a single mitochondrion (Tyler et al., 2001). We show that the extreme N-terminal region of *Tb*PEX11 contains a cryptic N-terminal signal that directs PEX11 to the mitochondrion if its glycosomal transport is blocked.

## 2 Materials and methods

### 2.1 Cloning

*Escherichia coli*, yeast, and *Trypanosoma* expression plasmid constructs and cloning strategies are listed in Table 1, and oligonucleotide sequences are listed in Table 2. Point mutations in *Sc*PEX11, *Tb*PEX11_1-89aa,_ and the gene fragment deletions (*Tb*PEX11_1-76aa_ and *Tb*PEX11-GFP constructs) were generated by overlap extension PCR. Sequences of the constructs, mutations, and gene fragment deletions were verified for all constructs by automated Sanger sequencing.

**TABLE 1:** Strains and plasmids.

Sl no.	Expression in	Construct	Primer pair	Restriction sites	Cloned in vector
1	*E. coli*	GST-*Tb*PEX19	RE2926 - RE7038	BamHI/XhoI	pGEX4T-2
2	*E. coli*	GST-*Hs*PEX19	pAH5 Halbach et al. (2005)		
3	*S. cerevisiae*	GAL4 AD-*Sc*PEX14	Albertini et al. (1997)		
4	*S. cerevisiae*	GAL4 BD-*Sc*PEX17_167-199aa_	Girzalsky et al. (2006)		
5	*S. cerevisiae*	GAL4 AD-*Tb*PEX19_1-285aa_	RE3310 - RE3311 (pIA13, AG Erdmann)	SalI/NotI	pPC86
6	*S. cerevisiae*	GAL4 BD-*Tb*PEX11_1-218aa_	RE7303 - RE7306	SalI/NotI	pPC97
7	*S. cerevisiae*	GAL4 BD-*Tb*PEX11_1-89aa_	RE7303 - RE7305	SalI/NotI	pPC97
8	*S. cerevisiae*	GAL4 BD-*Tb*PEX11_90-218aa_	RE7304 - RE7306	SalI/NotI	pPC97
9	*S. cerevisiae*	GAL4 BD-*Tb*PEX11_1-76aa_	RE8882—RE8883	Quick change PCR	pPC97
10	*S. cerevisiae*	GAL4 BD-*Tb*PEX11_1-89aa_ (S 25 D)	RE7713 - RE7714	Quick change PCR	pPC97
11	*S. cerevisiae*	GAL4 BD-*Tb*PEX11_1-89aa_ (S 25 P)	RE7715 - RE7716	Quick change PCR	pPC97
12	*S. cerevisiae*	GAL4 BD-*Tb*PEX11_1-89aa_ (L 31 P)	RE7717 - RE7718	Quick change PCR	pPC97
13	*S. cerevisiae*	GAL4 BD-*Tb*PEX11_1-89aa_ (S 25 P, L 31 P)	RE7715 - RE7716, RE7717 - RE7718	Quick change PCR	pPC97
14	*S. cerevisiae*	GAL4 AD-*Hs*PEX19	RE7706 - RE7707	SalI/NotI	pPC86
15	*S. cerevisiae*	GAL4 BD-*Hs*PEX11_1-73aa_	RE7843 - RE7844	SalI/NotI	pPC97
16	*S. cerevisiae*	GAL4 BD-*Hs*PEX11_1-73aa_	RE7708 - RE7709	SalI/NotI	pPC97
17	*S. cerevisiae*	GAL4 BD-*Hs*PEX11_1-74aa_	RE7845 - RE7846	SalI/NotI	pPC97
18	*S. cerevisiae*	*Sc*PEX11-GFP	Boutouja et al. (2019)		
19	*S. cerevisiae*	*Sc*PEX11 (L 35 P)-GFP	RE8063 - RE8064	Quick change PCR	pUG35
20	*T. brucei*	*Tb*PEX11-GFP	RE8070 - RE8071	BstBI/BamHI	pGN1
21	*T. brucei*	*Tb*PEX11_Δ13-35aa_-GFP	RE8072 - RE8073	Quick change PCR	pGN1
22	*T. brucei*	*Tb*PEX11_Δ77-99aa_-GFP	RE8074 - RE8075	Quick change PCR	pGN1
23	*T. brucei*	*Tb*PEX11_1-90aa_-GFP	RE7378 - RE7379	ApaI/BamHI	pGN1
24	*T. brucei*	*Tb*PEX11_1-90aa-Δ13-35aa_-GFP	RE8072 - RE8073	Quick change PCR	pGN1
25	*T. brucei*	*Tb*PEX11_1-90aa-Δ2-11aa_-GFP	RE8096 - RE7379	BstBI/BamHI	pGN1

**TABLE 2:** Oligonucleotides.

Primer	Sequence 5′to 3′
RE2926	GAT​CGG​ATC​CAT​GTC​TCA​TCC​CGA​CAA​TGA​C
RE3310	GAT​CGT​CGA​CGA​TGT​CTC​ATC​CCG​ACA​ATG​AC
RE3311	GAT​CGC​GGC​CGC​CTA​CAC​TGA​TGG​TTG​CAC​ATC​G
RE7038	CCG​CTC​GAG​TTA​CAC​TGA​TGG​TTG​CAC​ATC​GGC​AAG​TCC
RE7303	TGG​ACC​GTC​GAC​GAT​GTC​TGA​GTT​CCA​AAG​GTT​TGT​T
RE7304	AAT​ATA​GTC​GAC​TAA​GTT​CCT​CCG​CGT​GCT​GTG​C
RE7305	AAG​ATA​GCG​GCC​GCT​TAC​AAG​ACC​TCT​TTC​ATG​TTG​AC
RE7306	AAT​AAG​CGG​CCG​CCT​ATT​TGA​TCT​TGT​TCC​AGT​TCA​A
RE7378	AAG​ATA​GGG​CCC​ATG​TCT​GAG​TTC​CAA​AGG​TTT​GTT
RE7379	AAA​TGG​ATC​CGA​TCC​GCT​TCC​CTT​CAA​GAC​CTC​TTT​CAT​GTT​GAC
RE7706	AAG​ACG​TCG​ACC​ATG​GCC​GCC​GCT​GAG​GAA​GGC​TG
RE7707	AAG​ACG​CGG​CCG​CTC​ACA​TGA​TCA​GAC​ACT​GTT​CA
RE7708	AAG​ATG​TCG​ACA​ATG​GAC​GCC​TGG​GTC​CGC​TTC​AG
RE7709	AAG​ACG​CGG​CCG​CTT​ATC​TTT​TGG​CTG​ACT​CAA​GG
RE7713	GCC​TTA​AAG​ACA​CCA​TCA​AAT​GCC​TTT​AGA​ATC​TTG​TCG​CGG​C
RE7714	GCC​GCG​ACA​AGA​TTC​TAA​AGG​CAT​TTG​ATG​GTG​TCT​TTA​AGG​C
RE7715	CTT​AAA​GAC​ACC​AGG​AAA​TGC​CTT​TAG​AAT​CTT​GTC​GCG​G
RE7716	CCG​CGA​CAA​GAT​TCT​AAA​GGC​ATT​TCC​TGG​TGT​CTT​TAA​G
RE7717	GTG​TCG​AGG​GAG​CCA​GGT​GCC​TTA​AAG​ACA​C
RE7718	GTG​TCT​TTA​AGG​CAC​CTG​GCT​CCC​TCG​ACA​C
RE7843	AGA​AGT​CGA​CAA​TGG​ACG​CCT​TCA​CCC​GCT​TCA​CC
RE7844	AAG​AGC​GGC​CGC​TTA​CTG​CTC​AGT​TGC​CTG​TAT​AG
RE7845	AAG​AAG​TCG​ACA​ATG​GCG​TCG​CTG​AGC​GGC​CTG​G
RE7846	AAG​AGC​GGC​CGC​TTA​TTG​CTT​AGT​GTA​GAC​AAA​CA
RE8063	CTG​CTA​AAA​ATC​TTG​CTG​GAT​ACT​GCA​GTA​ATC​TGA​GAA​CCT​TTT​CTC​TGC
RE8064	GCA​GAG​AAA​AGG​TTC​TCA​GAT​TAC​TGC​AGT​ATC​CAG​CAA​GAT​TTT​TAG​CAG
RE8070	AAG​AAT​TCG​AAA​TGT​CTG​AGT​TCC​AAA​GGT​TTG​TT
RE8071	AAG​ACG​GAT​CCG​ATT​TGA​TCT​TGT​TCC​AGT​TCA​A
RE8072	TTC​TTG​AGA​CCT​GTC​AGA​GCC​GCT​CAA​G
RE8073	GAC​AGG​TCT​CAA​GAA​GCT​TAA​CAA​ACC​TTT​GG
RE8074	TTC​AGG​ATT​ATG​TGC​TCG​GCG​ACA​ATG
RE8075	GCA​CAT​AAT​CCT​GAA​TGG​CAT​TCT​GC
RE8096	AAG​ACT​TCG​AAA​TGG​AGC​AGA​CAG​ATG​GCC​GCG​AC
RE8882	TTC​AGG​ATT​AAG​CGG​CCG​CTA​AGT​AAG
RE8883	CCG​CTT​AAT​CCT​GAA​TGG​CAT​TCT​GCA​TC

### 2.2 Cell culture

#### 2.2.1 *Trypanosoma*

*Trypanosoma* procyclic form (PCF) 29–13 cell line (co-expressing T7 RNAP and TetR) was used in this study. PCF cells were grown in SDM-79 medium supplemented with 10% FBS at 28°C (Brun and Schonenberger, 1979; Krishna et al., 2023). PCF cultures were maintained at 1 × 10^6^–30 × 10^6^ cells/mL. Transfections were performed with NotI-linearized plasmid constructs (pGN1-*Tb*PEX11 constructs), which was genomically integrated into the rRNA locus in the genome of cell line 29–13. Clones were selected using Blasticidin (10 μg/mL) as described previously (Kalel et al., 2015).

#### 2.2.2 Yeast

*S. cerevisiae* wild-type strain BY4742 (for microscopy) and strain PCY2 (WT or Δ*pex19* for yeast two-hybrid assay) were grown in double dropout SD synthetic media as described in section 2.3 and 2.4.2. Yeast cells were transformed by the traditional Lithium-acetate method (Gietz and Woods, 2002).

#### 2.2.3 *Escherichia coli*

*Escherichia coli* strain TOP10 was used for all plasmid amplifications and BL21 (DE3) strain was used for heterologous expression of recombinant GST-PEX19 fusion proteins. Liquid *E. coli* cultures were grown at 37°C under continuous shaking in LB medium containing the appropriate selective antibiotic (100 μg/mL Ampicillin).

### 2.3 Yeast two-hybrid analysis (Y2H)

Y2H studies were performed based on the Yeast protocols handbook (Clontech, Protocol No. PT3024-1, Version No. PR742227). Full length or various truncations of *Trypanosoma* or Human PEX11 were cloned in pPC97 vector containing GAL4-DNA Binding Domain (BD) and full-length *Trypanosoma* or human PEX19 were cloned in pPC86 vector containing GAL4-Activation domain (AD), as described in Table 1. Co-transformation of various two-hybrid plasmids i.e., BD and AD constructs were performed in WT PCY2 or Δ*pex19* PCY2 strain in case of *Hs*PEX19-*Hs*PEX11 constructs. The clones were selected on SD synthetic medium without tryptophan and leucine. A filter-based β-galactosidase assay and liquid culture assay using ONPG were performed in three replicates as described in the Yeast protocols handbook (Clontech).

### 2.4 Microscopy

#### 2.4.1 *Trypanosoma*

*Trypanosoma* stable cell lines (Procyclic 29:13) encoding various tetracycline inducible PEX11-GFP constructs (full-length and mutants) were induced with 1 μg/mL tetracycline or treated with DMSO alone as negative control. Cells were sedimented and fixed by resuspension in 4% paraformaldehyde in PBS (phosphate-buffered saline, pH 7.4) at 4°C for 15 min. Fixed *Trypanosoma* cells were washed two times with PBS and stored at 4°C in a dark box. For imaging, fixed cells were immobilized on a glass slide (StarFrost 76 × 26 mm, Knittel Glass) pre-coated with 10% (v/v) of poly-L-lysine (Sigma-Aldrich) in water for 1 h at room temperature (RT). Further, the cell membranes were permeabilized with PBS containing 0.125% Triton X-100 and incubated for 10–15 min, followed by blocking with PBS containing 3% BSA, 0.25% Tween-20 for 1 h at RT. Rabbit α-Aldolase antibody (1:500 in blocking buffer) was used as glycosomal marker and incubated at RT for 1 h. Following 5 washes with PBS, anti-rabbit Alexa fluor 594 secondary antibodies (1:200 dilution) in PBS was applied and incubated for 30 min at RT in the dark. Further, the stained samples were washed, dried, and layered with anti-fading mounting medium, i.e., Mowiol with DAPI (4′,6-diamidino-2-phenylindole).

For mitochondrial staining, tetracycline-induced or uninduced (DMSO-treated) *Trypanosoma* cells were harvested and resuspended in the culture medium containing 75 nM MitoTracker® Deep Red and incubated for 5 min at 28°C. Following incubation, cells were washed with PBS twice and resuspended in the culture medium and further incubated for 30 min at 28°C. Subsequently, cells were fixed with 4% paraformaldehyde in PBS, and samples were prepared for microscopy as mentioned above.

Glycosome- or mitochondrion-stained cells were visualized and imaged with a Zeiss Elyra microscope and were analyzed using Zeiss Zen 3.2 software (blue edition). Both aldolase and MitoTracker which are markers for glycosome and mitochondrion respectively, are pseudo-colored to magenta for visualization.

#### 2.4.2 Yeast

BY4742 yeast strain co-transformed with the plasmids encoding *Sc*PEX11-GFP (WT (Boutouja et al., 2019) and L_35_ to P mutant, with the endogenous promoter) and DsRed-SKL (as a peroxisomal reporter (Kuravi et al., 2006)) were grown overnight (16 h) with shaking in an SD synthetic medium without uracil and histidine. Next day the precultures were diluted to 0.1 OD_600_/mL and were incubated under shaking until the cell density reached 0.6–0.8 OD_600_/mL. After incubation, 1–2 mL cultures were harvested and washed with water. For mitochondrial staining, 5 mL of yeast cells, grown to a density of 0.6–0.8 OD_600_/mL, expressing the *Sc*PEX11 constructs were stained with 150 nM MitoTracker™ Orange CMTMRos (Invitrogen) for 30 min with shaking in dark. Following incubation, 1–2 mL cultures were harvested and washed with water. All incubation steps were performed at 30°C. Yeast cells expressing various fluorescent proteins were directly visualized microscopically without fixation. Microscopy was performed with Carl Zeiss Microscope, using the Axiovision 4.6.3 software, and images were analyzed using Zen 3.2 (blue edition), a Carl Zeiss software. The DsRed-SKL, a peroxisomal reporter is pseudo-colored to magenta for visualization.

### 2.5 Peptide array

The immobilized peptides of 15-amino acids length, sequentially overlapping by 13 residues (2aa shift), representing the entire sequence of *Tb*PEX11 or the N-terminal domains of three human PEX11 isoforms were synthesized on a cellulose membrane as described previously (Hilpert et al., 2007; Neuhaus et al., 2014). The peptide array was first washed with ethanol for 10 min with gentle shaking followed by three washes with TBS (50 mM Tris, 137 mM NaCl, 2.7 mM KCl, adjusted to pH 8) for 10 min each. Further, the peptide array was incubated with a blocking buffer (TBS +3% BSA +0.05% Tween-20) for 2 h at room temperature (RT). The purified recombinant proteins GST-*Tb*PEX19, GST-*Hs*PEX19, or GST alone (10 mL of 1 µM solution prepared in blocking buffer) were incubated with the arrays for 1 h at 4°C. Then, the arrays were washed three times for 10 min at RT with TBS, and subsequently incubated with the anti-GST monoclonal antibody (Sigma, 1:2000) at RT for 1 h. Followed by three washes with TBS (10 min each), a secondary antibody (Horseradish peroxidase-coupled anti-mouse IgGs, 1:5,000 in blocking buffer) was applied, and the array was further incubated for 1 h at RT. After three washes with TBS, the array was scanned with chemiluminescence substrate (WesternBright Sirius) using Azure sapphire biomolecular imager.

### 2.6 Protein expression and purification, *in vitro* pull-downs and AlphaScreen binding assay

#### 2.6.1 Protein expression and purification

The expression plasmids pGEX4T2, pGEX4T2-*Tb*PEX19 or pGEX4T1-*Hs*PEX19, encoding for GST, GST-*Tb*PEX19 or GST-*Hs*PEX19, respectively, were transformed into BL21 (DE3) *E. coli* strain. Single colonies were inoculated in LB medium containing ampicillin and incubated overnight with shaking at 37°C. On the following day, the cultures were reinoculated with 0.1 OD_600_/mL and further incubated at 37°C with shaking, until the cell density reached 0.6 OD_600_/mL. Protein expression was induced with 1 mM IPTG for 4 h at 30°C. Harvested cell pellets were stored at −20°C before use. For protein purification, *E. coli* cell pellets were resuspended in PBS with protease inhibitors (5 μg/mL Antipain, 2 μg/mL Aprotinin, 0.35 μg/mL Bestatin, 6 μg/mL Chymostatin, 2.5 μg/mL Leupeptin, 1 μg/mL Pepstatin, 0.1 mM PMSF, 25 μg/mL DNAse and 1 mM DTT). Cells were disrupted using EmulsiFlex and unbroken cells were removed by centrifugation at 4,500 rpm for 15 min (rotor SX4400, Beckman Coulter). The resulting supernatant (SN1) was subjected to a high-speed centrifugation at 14,000 rpm for 1 h (rotor SS-34, Thermo Scientific), which yielded supernatant 2 (SN2), a soluble fraction that included overexpressed proteins. Proteins were purified by affinity chromatography using Glutathione Agarose 4B beads (Macherey-Nagel). To this end, SN2 was incubated with the pre-equilibrated glutathione agarose beads for 2 h in a tube rotator. After collection of the flow-through, using a gravity flow column, the protein-bound beads were washed five times with PBS. Proteins were eluted with 10 mM reduced glutathione in 50 mM Tris-Cl (pH 8). The buffer of the eluted protein was exchanged to PBS using Amicon centrifugation tubes with molecular weight cut-off (MWCO) 10 kDa. The concentration of the proteins was determined by the Bradford method (Thermo, Coomassie Plus assay kit), and protein aliquots were stored at—80°C. All the purification steps were performed at 4°C.

#### 2.6.2 *In vitro* pull-down

20 µL bed volume Glutathione Agarose 4B beads (Macherey-Nagel) were incubated with 200 µg of recombinantly purified GST and GST-*Tb*PEX19 proteins in separate tubes for 2 h at 4°C with gentle rotation. Following incubation, beads were washed with PBS to remove unbound proteins. Subsequently, 25 µg of C-terminally His_6_-tagged synthetic peptides of crude grade, containing the corresponding *Tb*PEX19 binding regions in *Tb*PEX11 (BS1-BS3) were loaded to the respective tubes and were incubated for 2 h at 4°C with gentle rotation to allow binding of the peptides to GST-*Tb*PEX19 or control GST. Following washes with PBS, the bound proteins/peptides were eluted with 50 µL 10 mM reduced glutathione in 50 mM Tris (pH 8). The eluted samples were analyzed by SDS-PAGE followed by Coomassie staining and immunoblotting. The sequences of the *Tb*PEX11 peptides used for the pull-down are as follows, BS1: QTDGRDKILKAFSGVFKALGSLD-GS-His_6_, BS2: CRAKGKVNMKEVLKFLRVLCNFL-GS-His_6_ and BS3: VLDVVALYGALQKRASDPATS-GS-His_6_.

#### 2.6.3 AlphaScreen binding assay

N-terminal GST tagged *Tb*PEX19 and *Tb*PEX11 peptides with C-/N-terminal His_6_ were used for the interaction study with the AlphaScreen system. The final reaction volume used for the study was 25 μL, which consist of 5 μL of each protein solution (30 nM for PEX19 and 300 nM of PEX11 peptides), 5 μL of buffer, and 5 μL of solution for each of the donor and acceptor beads (5 μg/mL). The above solutions were prepared in reaction buffer [0.5% BSA v/v, PBS (pH7.4)] on the day of the assay. Compounds were incubated with the proteins for 30 min at room temperature (RT). 5 μL of AlphaScreen Nickel-chelate acceptor beads (cat. no. 6760619C, PerkinElmer^®^) were added to the above mixture following 15 min incubation at RT. 5 μL AlphaScreen Glutathione donor beads (cat. no. 6765300, PerkinElmer^®^) were added to the mixture. The complete 25 μL reaction solutions were incubated for 45 min at RT in the dark, and Alpha signals were captured with Cytation 5 plate reader (BioTek^®^) with the gain value set at 180. All above concentrations mentioned for the AlphaScreen assays were final concentrations unless otherwise stated. The sequences of the *Tb*PEX11 peptides used for the AlphaScreen assay are as follows, BS1: QTDGRDKILKAFSGVFKALGSLD-GS-His_6_, His_6_-GS-QTDGRDKILKAFSGVFKALGSLD and BS2: CRAKGKVNMKEVLKFLRVLCNFL-GS-His_6_. The binding assay were performed in three biological replicates, with 3 technical replicates each.

### 2.7 Immunoblotting

Proteins separated by SDS-PAGE were transferred on a nitrocellulose membrane with a pore size of 0.45 μm (Amersham Biosciences). Blotting was performed by using the MiniProtean III cell (BioRad) with blot transfer buffer (Dunn carbonate buffer) for 1 h with a constant current of 300 mA per chamber. Further, the membrane was blocked for 1 h at room temperature (RT), under constant swirling with 3% BSA in blot washing buffer (TBS with 0.05% Tween-20) to avoid nonspecific binding of antibodies. Then, the membrane was washed three times for 5 min at RT, and subsequently incubated with the primary antibodies in blot washing buffer at 4°C overnight. Following primary antibodies were used in this study: mouse anti-GFP (Sigma, 1:2,000), anti-GAL4 AD/-BD (Santa Cruz Biotechnology, 1:1,000) or anti-His_6_ (Invitrogen, 1:2,000); rabbit anti-*Trypanosoma* Aldolase (1:20,000) or Enolase (1:20,000), and anti-Porin (*S. cerevisiae*, 1:10,000). After three washing steps, the corresponding secondary antibodies i.e., goat anti-rabbit IRDye 680 or goat anti-mouse IRDye 800CW (LI-COR Biosciences, both 1:15,000 in blot wash buffer) were applied, and the membrane was further incubated for 30 min at RT in the dark. Following three washes, immunoblots were scanned using the Li-Cor Odyssey 9120 Infrared Imaging System.

### 2.8 Statistical analysis

Microscopic data was collected from two independent *S. cerevisiae* or *T. brucei* cultures. Images were quantified using Pearson’s correlation coefficient, which was calculated with colocalization tool of Zen 3.6 pro (blue edition). Statistical significances for colocalization studies were calculated using a one-way ANOVA (mixed) by Dunnett’s multiple comparisons test (comparison with WT control) with each row representing matched or repeated measures. Statistical analysis for AlphaScreen results was done using two-way ANOVA with Bonferroni’s multiple comparison test (comparison with respective controls) with the values obtained from three independent biological replicates, each with three technical replicates.

## 3 Results

### 3.1 Identification and validation of PEX19 binding sites in *Trypanosoma brucei* PEX11

**FIGURE 1:**
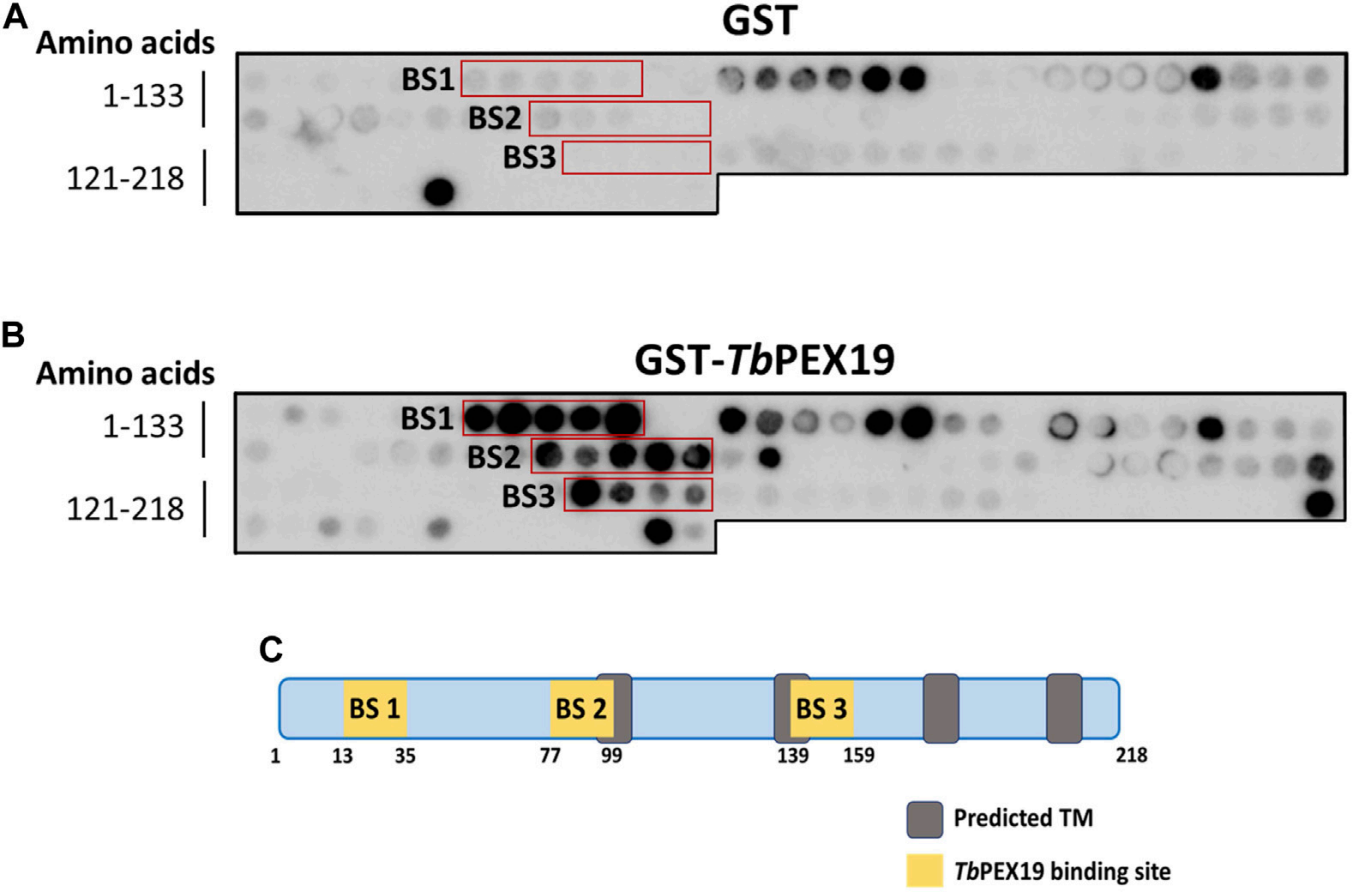
Identification of PEX19 binding sites in *Trypanosoma* PEX11 using synthetic peptide arrays. Synthetic 15-mer peptides with 2-amino acids shifts corresponding to the complete *Tb*PEX11 protein sequence were synthesized on cellulose membrane and probed with GST as negative control **(A)** or GST-*Tb*PEX19 **(B)**. Bound analyte was immuno-detected using primary antibodies against GST and horseradish peroxidase coupled secondary antibodies, followed by the signal detection using chemiluminescence. Three regions in *Tb*PEX11 showed clear and specific interaction with *Tb*PEX19 as compared to the GST control (red boxes, marked BS1-BS3). **(C)** Scheme of *Tb*PEX11 showing the identified binding regions in relation to transmembrane segments predicted using Phobius webtool (https://phobius.sbc.su.se/) (Supplementary Figure S2).

PEX19 acts as a cytosolic chaperone and receptor for the import of newly synthesized class 1 peroxisomal membrane proteins (PMPs), except the class II PMP i.e., PEX3, which can be imported independent of PEX19 (Sacksteder et al., 2000; Jones et al., 2004). PMPs contain multiple PEX19 binding sites, which are well characterized in yeast and humans. This includes the most abundant yeast PMP Pex11p and related PEX11-family proteins Pex25p and Pex27p (Rottensteiner et al., 2004; Halbach et al., 2005). In trypanosomatid parasites, PEX19 binding sites have been identified in various glycosomal membrane proteins as well as parasite specific PEX11 family proteins GIM5A/B (Saveria et al., 2007). However, the PEX19-binding sites (-BS) in parasite PEX11 remained uncharacterized. To identify these binding sites, we obtained synthetic peptide arrays, containing consecutive 15-amino acid peptides with two amino acid shifts, representing the entire sequence of *Tb*PEX11. Affinity purified recombinant GST-*Tb*PEX19 (Purification profile described in Supplementary Figure S1), or GST alone were incubated with the arrays and bound proteins were immuno-detected using monoclonal anti-GST antibodies. Immunodetection of at least three consecutive spots were considered as potential PEX19 binding sites (Rottensteiner et al., 2004). Comparison of control and test peptide arrays revealed the presence of three potential PEX19 binding sites (BS1-BS3) in *Tb*PEX11 (Figure 1). The topological prediction of transmembrane domains (TMDs) using Phobius webserver (Kall et al., 2007) indicates that *Tb*PEX11 contains four TMDs and an N-terminal extension of about 90 amino acids to the cytosol (Supplementary Figure S2). The first PEX19 binding site (BS1) is present close to the N-terminus of *Tb*PEX11 between amino acid (aa) residues 13–35, the second and third PEX19 binding sites are located between aa77-99 and aa139-159, respectively, in proximity of the first and second predicted transmembrane domains (Figure 1C). Both N- and C-termini of *Tb*PEX11 face the cytosol (Lorenz et al., 1998), which implies that the BS1 would remain exposed to the cytosol even after targeting and insertion of *Tb*PEX11 into the glycosomal membrane.

PEX19 binding motifs are conserved between peroxisomal proteins of yeast or mammals and trypanosomal glycosomal proteins (Saveria et al., 2007). Probing of the *Tb*PEX11 peptide array with GST-tagged recombinant human PEX19 also revealed a similar binding pattern (Supplementary Figure S3) as observed with *Tb*PEX19 (Figure 1). This further demonstrates the conservation of PEX19-BSs, which can be recognized by PEX19 from different organisms.

**FIGURE 2:**
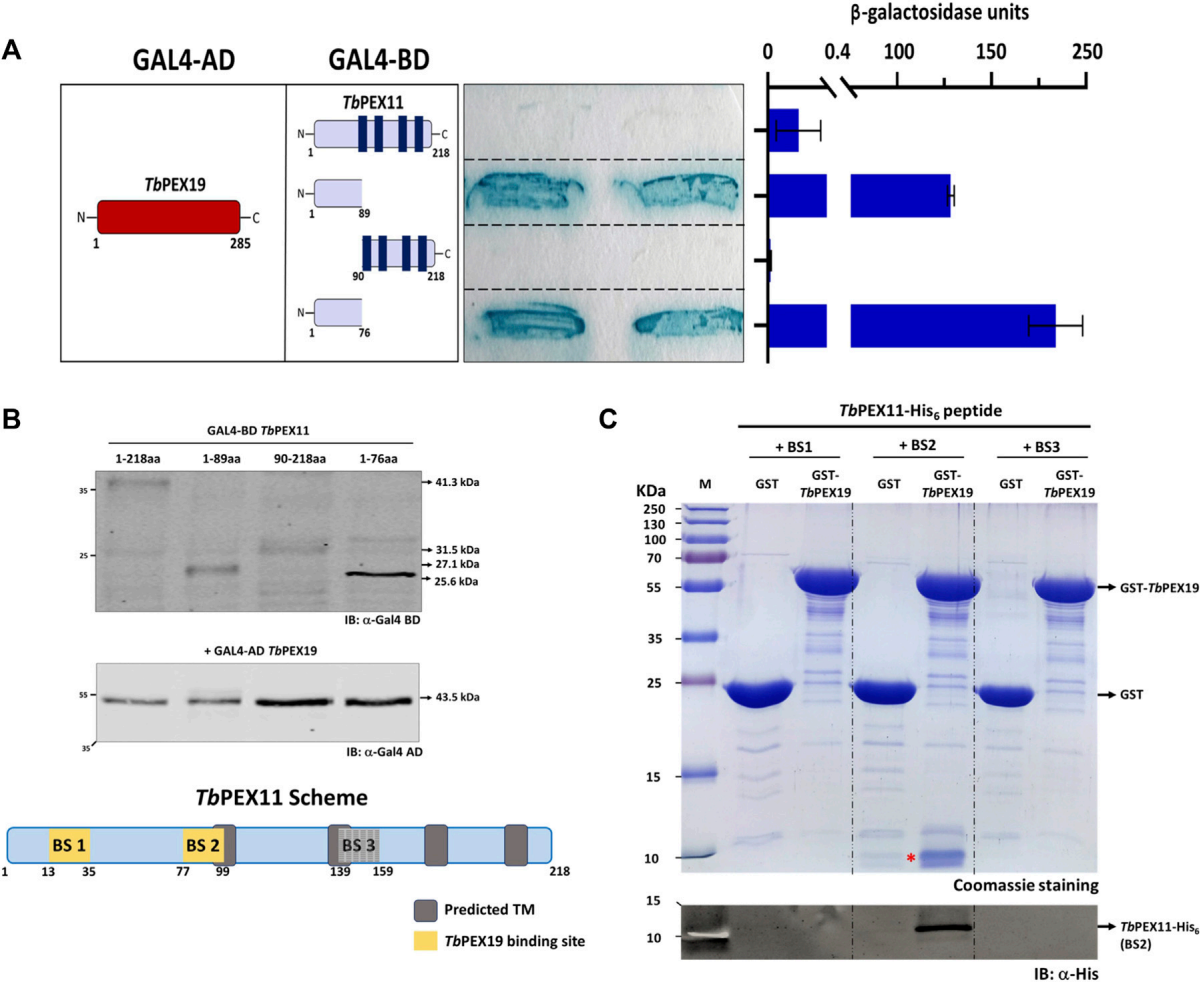
Validation of *Tb*PEX19 binding sites in *Tb*PEX11 **(A)** Yeast two-hybrid assay (Y2H): Full length *Tb*PEX19 and various *Tb*PEX11 constructs fused to the GAL4-activation domain (AD) or -binding domain (BD) as indicated (left panel) were co-transformed into PCY2 yeast strain and analyzed by Colony-lift filter assay (middle panel) and liquid ONPG assay (right panel). Both assays were done in three replicates and the β-galactosidase activity units shown are an average of the technical replicates with three different clones. Error bars represent mean with standard deviations. The soluble N-terminal fragments of *Tb*PEX11 comprising BS1 and partially BS2 (1–89) as well as a shorter fragment containing only BS1 (1–76) showed a clear interaction with full-length *Tb*PEX19. Full length *Tb*PEX11 showed a weaker interaction, while the PEX11-fragment (90–218), lacking the N-terminal PEX19 BSs, did not interact with *Tb*PEX19. The GAL4-AD fusion of *Tb*PEX19 and the various GAL4-BD fusions of *Tb*PEX11 were tested for autoactivation and no coloration of the filter was seen (not shown). **(B)** Expression of the GAL4-AD and -BD fusion proteins in the yeast two-hybrid (Y2H) assay was confirmed by immunoblotting with monoclonal antibodies against GAL4-AD and -BD as indicated. Arrow marks on the right correspond to the predicted molecular weights of the fusion proteins. The scheme at the bottom shows the modular structure of *Tb*PEX11, highlighting the identified putative *Tb*PEX19 binding regions. **(C)** *In vitro* pull-down of *Tb*PEX19 with His_6_-tagged synthetic peptides of *Tb*PEX11 that correspond to the binding regions highlighted in the PEX11 scheme. Recombinant GST-*Tb*PEX19 or GST as negative control were pre-incubated with Glutathione agarose beads, followed by incubation with the C-terminally His_6_-tagged synthetic peptides of *Tb*PEX11. After thorough washing, bound proteins were eluted with reduced glutathione and analyzed by SDS-PAGE and staining with Coomassie brilliant blue (upper panel). The peptide corresponding to BS2 (migrating at ∼10 kDa and marked with a red asterisk) was pulled down with GST-*Tb*PEX19, which was also confirmed by immunoblotting using an anti-His monoclonal antibody (lower panel). The putative BS3 could not be validated by either of the assays, therefore it was not considered further and is shown as grey box in the scheme in **(B)**. AD: Activation domain, BD: Binding domain, ONPG: ortho-Nitrophenyl-ß-galactoside.

Binding of *Tb*PEX19 to the newly identified regions in *Tb*PEX11 were further investigated by yeast two-hybrid (Y2H) analysis and *in vitro* binding assays using pull-down and AlphaScreen. For the Y2H assay, GAL4-AD fusion of *Tb*PEX19 was used since the corresponding GAL4-BD fusions showed autoactivation (not shown). In addition to this, the corresponding GAL4-BD fused PEX11 constructs did not result in the auto activation when tested with GAL4-AD alone (not shown). Various *Tb*PEX11 constructs fused to GAL4-BD were tested for interaction with *Tb*PEX19 (Figure 2A). Full length *Tb*PEX11 showed only a very weak interaction with *Tb*PEX19. This could be due to the presence of several predicted transmembrane domains in *Tb*PEX11, which may hinder the translocation into the nucleus and activation of GAL-promoter. However, the N-terminal fragment of *Tb*PEX11_1-89aa_ that has been predicted to be soluble (Supplementary Figure S2) showed a clear and strong interaction with the full length *Tb*PEX19, in both plate-based (sensitive) and liquid Y2H assays (quantitative) (Figure 2A). Construct that lacks the N-terminal domain but contains BS3 (*Tb*PEX11_90-218aa_) did not interact with *Tb*PEX19. *Tb*PEX11_1-89_ contains BS1 as well as partial BS2. We also tested a shorter construct that contains only BS1 (*Tb*PEX11_1-76_), which still showed a strong interaction with *Tb*PEX19. Immunoblotting confirmed that all constructs are expressed in yeast at correct molecular weights (Figure 2B). Furthermore, we opted to introduce two mutations in *Trypanosoma* PEX11. The first mutation replaced serine 25, which is in the *Tb*PEX19 BS1 region, by aspartate to mimic phosphorylation (based on the post-translational modifications database). For the second mutation, we referred to a study in baker’s yeast, which showed that replacing leucine at position 35 by proline results in the loss of interaction (Rottensteiner et al., 2004). We aligned the sequence with *Sc*PEX11 and chose the closest leucine residue (position 31) for replacement by proline. Mutation of serine_25_ to aspartate i.e., phospho-mimicking did not affect the interaction, whereas mutation of serine_25_ or leucine_31_ individually and together to proline within BS1 in *Tb*PEX11_1-89_ led to a reduced or complete abolishment of the interaction with *Tb*PEX19 (Supplementary Figure S4). Based on the peptide blot and the Y2H studies, it can be concluded that BS1 is a *bona fide* PEX19 binding motif.

As an alternative, we obtained C-terminally His_6_-tagged synthetic peptides of *Tb*PEX11 corresponding to the three putative PEX19 binding sites. Affinity pull-down was performed with GST-*Tb*PEX19 or GST alone as negative control, which were bound to the glutathione affinity beads. Glutathione eluates of the *in vitro* pull-downs were analyzed by Coomassie staining as well as immunoblotting using anti-His_6_ tag antibodies (Figure 2C; full profile of the pull-downs is shown in Supplementary Figure S5). The analysis shows that the synthetic peptide corresponding to BS2 (running at ∼10 kDa) is efficiently retained with *Tb*PEX19 but not with GST alone. Similar binding of the BS2 representing peptide of *Tb*PEX11 was also observed with recombinant human GST-PEX19 (not shown). The synthetic peptides corresponding to BS1 and BS3 (both running at ∼10 kDa) did not bind to recombinant GST-*Tb*PEX19 in this assay. The third putative PEX19 binding site (BS3) in *Tb*PEX11 that was identified in the peptide array analysis (Figure 1) could not be further validated by the methods employed here and was not considered further.

Finally, we analyzed the interaction of BS1 and BS2 with *Tb*PEX19 with the more sensitive AlphaScreen assay. This assay was performed using C-terminally His_6_-tagged peptides that represent the *Tb*PEX11 binding sites with GST-*Tb*PEX19 or GST as negative control (Supplementary Figure S6). Again, the BS2 showed a clear interaction with *Tb*PEX19, while the BS1 did not interact. As the interacting BS1 containing region was N-terminally tagged in the Y2H assay (Figure 2A), we considered that the orientation of the tag might have an influence and therefore analyzed the interaction of an N-terminally tagged BS1-peptide, which indeed showed a significant interaction with *Tb*PEX19 (Supplementary Figure S6). Taken together this study identified two PEX19 binding sites in *Tb*PEX11 (BS1 and BS2).

### 3.2 Role of PEX19 binding sites in glycosomal targeting of *Tb*PEX11

**FIGURE 3:**
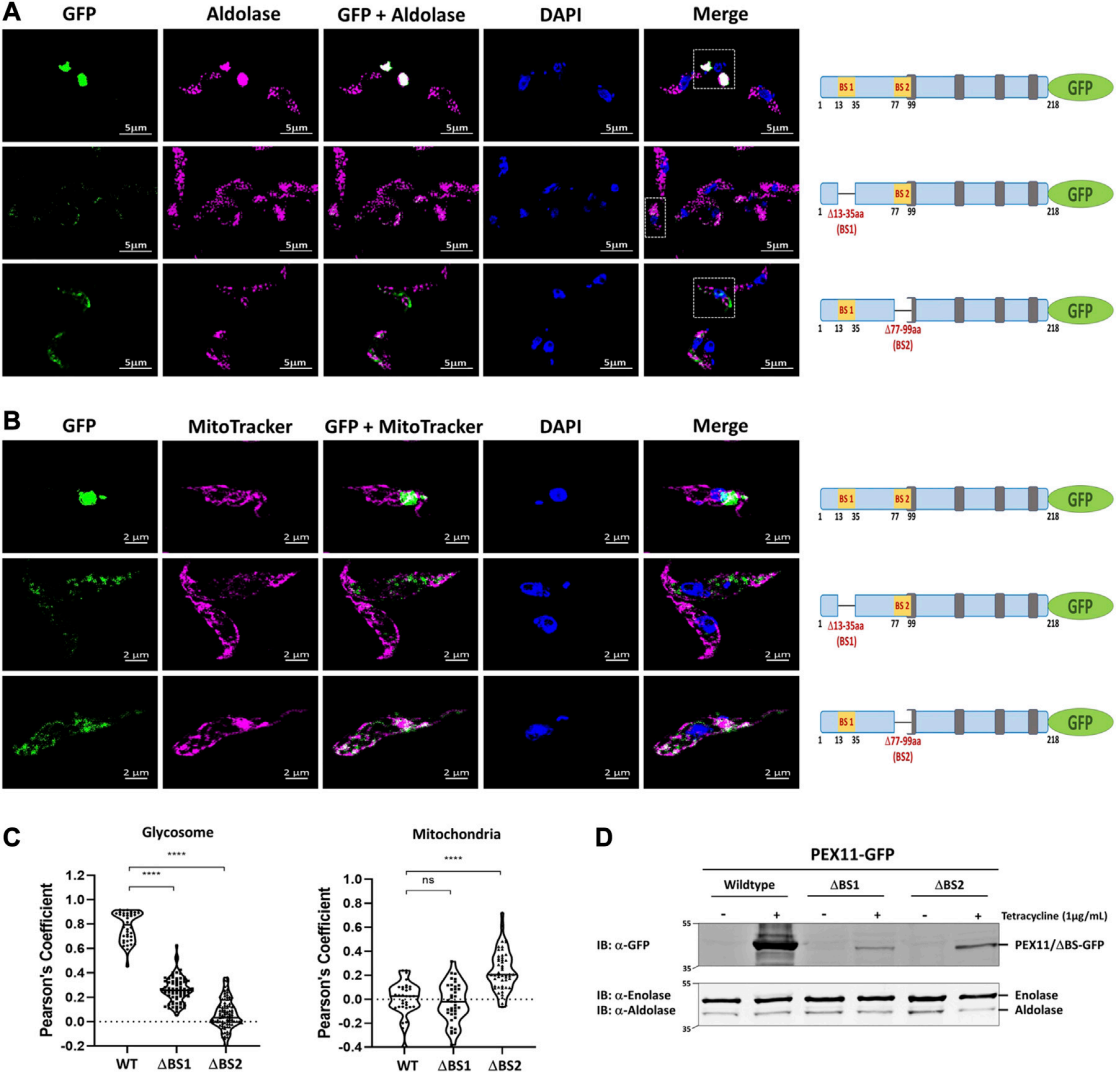
PEX19 binding sites are required for maintenance and essential for glycosomal targeting of *Tb*PEX11. **(A)** Binding site 2 (BS2) is required for glycosomal targeting of *Tb*PEX11. *Trypanosoma brucei* parasites (procyclic form) expressing tetracycline-induced and C-terminally GFP tagged *Tb*PEX11 constructs (wildtype or mutant proteins lacking PEX19 binding sites) were analyzed for localization of the GFP fusion proteins, the glycosomal marker aldolase, as well as the DAPI-stained nucleus and kinetoplast by fluorescence- or immunofluorescence microscopy. The GFP fusion of wildtype *Tb*PEX11 (upper panel) did colocalize with the glycosome marker aldolase (pseudo-colored to magenta). It is also evident that the overexpression of the full-length *Tb*PEX11 results in the clustering of glycosomes as previously reported (Lorenz et al., 1998). The mutant lacking the first PEX19 binding site (middle panel) partially colocalized with the glycosome marker aldolase. In this case, a clustering of glycosomes was not seen, most likely as the steady-state concentration of the truncated protein was much lower than the corresponding full-length *Tb*PEX11 (see below). PEX11-GFP harboring deletion of BS2 (Δ77-99aa) did not colocalize with the glycosomal marker aldolase (lower panel), but instead showed mislocalization to mitochondrion as demonstrated by colocalization with the mitochondrial marker MitoTracker (pseudo-colored to magenta) **(B)**. Scale bar—5 μm and −2 μm. Schematic representation of the various PEX11-GFP constructs is shown on the right. **(C)** Quantification of the colocalization to glycosomes (left) or mitochondrion (right). The Pearson’s coefficient of colocalization to respective organelle is shown. Dots within the violin plot indicates individual Pearson correlation coefficient data points and the central line represents the median. Statistical significance were calculated by one-way ANOVA, with Dunnett’s multiple comparisons test (n ≥ 35 cells). ****, *p* < 0.0001; ns, not significant. **(D)** Analysis of the expression levels of PEX11-GFP (wildtype and mutants) upon tetracycline induction (+/−) by immunoblotting with anti-GFP antibodies. Cytosolic marker enolase and glycosomal marker aldolase served as the loading controls (lower panel). Wildtype *Tb*PEX11 expression was highly induced, resulting in a high steady-state concentration, while the steady-state concentration of both truncation mutants of *Tb*PEX11 were very low in comparison to the wildtype *Tb*PEX11, most likely due to an instability of the *Tb*PEX11 constructs lacking either of the PEX19 binding sites.

We performed immunofluorescence microscopy analysis to assess the relevance of the newly identified PEX19 binding sites for the topogenesis of *Tb*PEX11. Tetracycline inducible stable cell lines of *Trypanosoma* were generated, which express C-terminally GFP-tagged full-length *Tb*PEX11 and variants lacking either BS1 or BS2. Glycosomal localization of the constructs was investigated by analysis of colocalization of the fluorescent GFP-fusions of *Tb*PEX11 with the glycosomal marker enzyme aldolase, which was monitored by immunofluorescence microscopy. Overexpressed *Tb*PEX11_WT_-GFP colocalized with the glycosomal marker, indicative for its glycosomal localization (Figure 3A, upper panel). However, frequently glycosomes appeared to cluster, confirming an earlier study reporting that overexpression of *Tb*PEX11 results in clustering of glycosomes in bloodstream form of *T. brucei* (Lorenz et al., 1998). The GFP fluorescence of cells expressing both truncated *Tb*PEX11 variants was much weaker in comparison to the wild-type protein and clustering of glycosomes was not seen. This is explained by the decreased steady-state concentration of both truncated proteins, which is much lower in comparison to the full-length *Tb*PEX11 as indicated by the corresponding immunoblots. (Figure 3D). However, the fluorescence was bright enough to allow investigation of their subcellular localization. *Tb*PEX11 lacking BS1 (*Tb*PEX11_△BS1_-GFP) still showed a partial glycosomal localization (Figure 3A, Middle panel), while the *Tb*PEX11 variant lacking BS2 (*Tb*PEX11_△BS2_-GFP) was mislocalized, as indicated by the lacking colocalization with the glycosomal marker (Figure 3A, lower panel). Taken together this result demonstrated that deficiency in either BS1 or BS2 affects the steady-state concentration of *Tb*PEX11. Thus, binding of PEX19 to either of these sites might stabilize the protein. This is in agreement with studies in yeast, which showed that various PMPs, including PEX11, are unstable and their steady state levels are significantly reduced in PEX19-or PEX3-deficient cells (Hettema et al., 2000). In the absence of BS1, the remaining small amount of *Tb*PEX11 is still directed to glycosomes, while in the absence of BS2, PEX11 is mistargeted, indicating that BS2 is essential for glycosomal targeting of *Tb*PEX11.

In yeast, PEX11 mislocalizes to mitochondria in cells lacking peroxisomal membranes (Hettema et al., 2000; Mattiazzi Usaj et al., 2015). To assess whether mislocalized *Tb*PEX11 is targeted to mitochondria also in trypanosomes, mitochondrial staining was performed. Indeed, colocalization of the truncated *Tb*PEX11 with the MitoTracker indicated that *Tb*PEX11 lacking BS2 is mistargeted to the mitochondrion (Figure 3B, lower panel).

**FIGURE 4:**
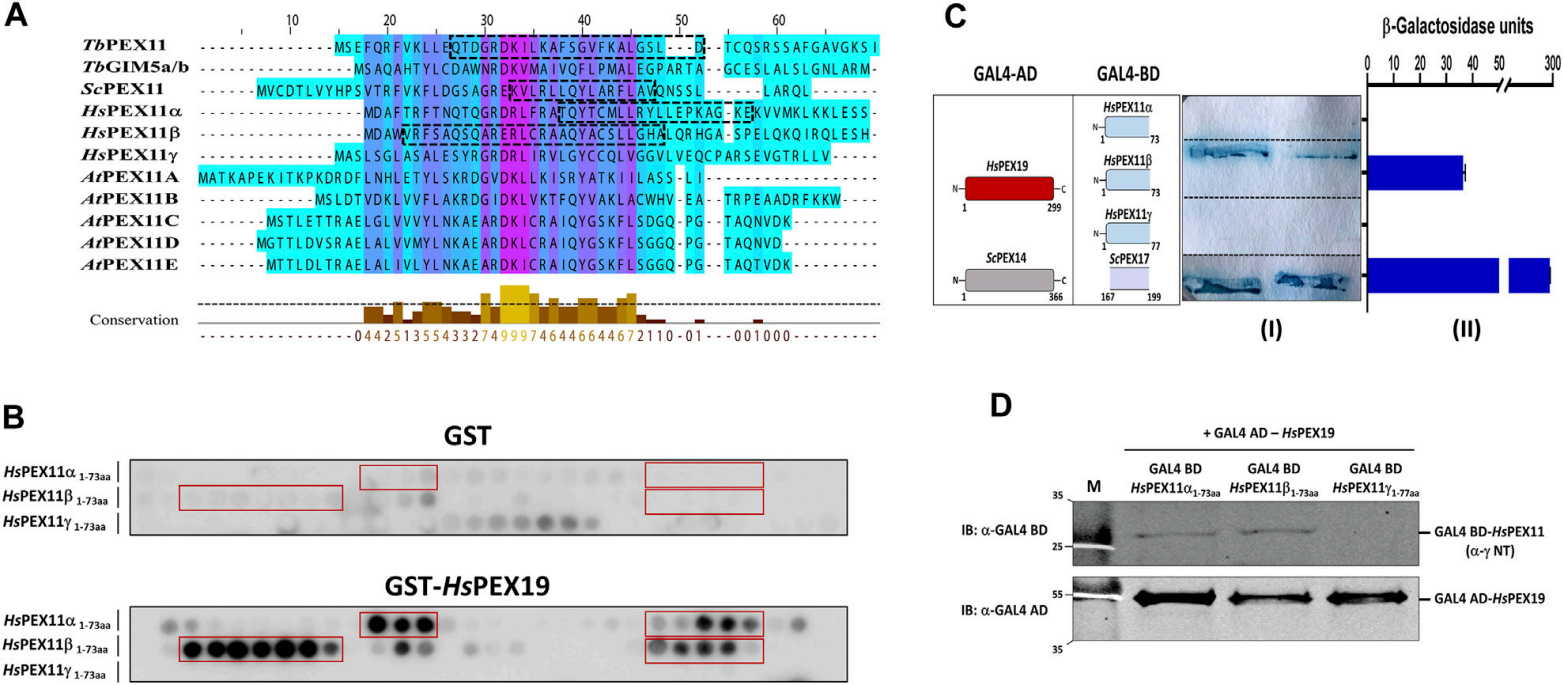
The N-terminal PEX19 binding site in PEX11 is conserved among species. **(A)** Multiple sequence alignment of N-terminal sequences of PEX11 proteins from parasites, baker’s yeast, humans, and plants shows a high degree of conservation. The N-terminal PEX19 binding site (BS1) identified in *Trypanosoma brucei* is indicated by a black dotted box. The known PEX19 binding site from yeast and the newly identified PEX19 binding sites in humans are also indicated by black dotted boxes. **(B)** Identification of N-terminal PEX19 binding site in the human members of the PEX11 family. Synthetic 15-mer peptides with 2-amino acids shift of the N-terminal protein sequence of *Hs*PEX11 (α, β and γ) were synthesized on cellulose membrane and probed with GST as a negative control (upper panel) or GST-*Hs*PEX19 (lower panel). The bound analyte was immunodetected using primary antibodies against GST and horseradish peroxidase coupled secondary antibody. The signal was monitored using chemiluminescence. Binding regions in *Hs*PEX11 (α and β) showed clear interaction with *Hs*PEX19 (red boxes). **(C)** Validation of identified binding sites by Yeast two-hybrid analysis using Δ*pex19* PCY2 strain. Scheme of the cotransformed *Hs*PEX19 and *Hs*PEX11 (α, β and γ) N-terminal constructs is shown on the left. Colony lift filter assay (I) and liquid ONPG assay (II) were performed using full-length *Hs*PEX19 and different constructs of *Hs*PEX11 fused to GAL4-AD and -BD as indicated. The interaction of yeast PEX14-PEX17 served as a positive control. Both assays were performed in three replicates and the β-galactosidase activity units shown are an average of the technical replicates with three different clones. Error bars represents mean with standard deviations. Of the three members of the PEX11-family only the N-terminal fragment of *Hs*PEX11β showed a clear interaction with full-length *Hs*PEX19. **(D)** Expression of the GAL4-AD and -BD fused proteins were tested by immunoblotting using GAL4-AD and -BD with monoclonal antibodies. No expression of Gal BD-*Hs*PEX11γ N-terminal fragment was detected.

Multiple sequence alignment of the N-terminal region comprising BS1 of *Trypanosoma*, yeast, human, and plant PEX11 family proteins or isoforms indicates a high degree of sequence conservation, suggesting that the region corresponding to trypanosomal BS1 is conserved among PEX11 species (Figure 4A). To investigate the capacity of this region of human PEX11 proteins for PEX19 binding, we obtained synthetic peptide array of N-terminal soluble domains of all three human PEX11 isoforms (15mer peptides with 2-amino acids shifts). The arrays were probed with GST alone as a negative control, which showed little or no background (Figure 4B, upper panel). Probing the array with GST-*Hs*PEX19 revealed that the peptides from PEX11γ did not bind PEX19, while PEX11α and PEX11β do contain potential PEX19-BS (Figure 4B, lower panel). To validate the interactions, Y2H analysis was performed to investigate the interaction of N-terminal domains of PEX11 isoforms and human PEX19 (Figure 4C). An interaction was seen only with PEX11β, in both the plate-based and the liquid assay. Immunoblot analysis of lysates of yeast cell used in Y2H shows that PEX11α and β were expressed, but not PEX11γ (Figure 4D). As PEX11γ was not expressed, no conclusion can be drawn from the negative result of the two-hybrid study. However, the results are clear in that PEX11β indeed does contain a PEX19 binding site in the region that corresponds to trypanosomal BS1.

**FIGURE 5:**
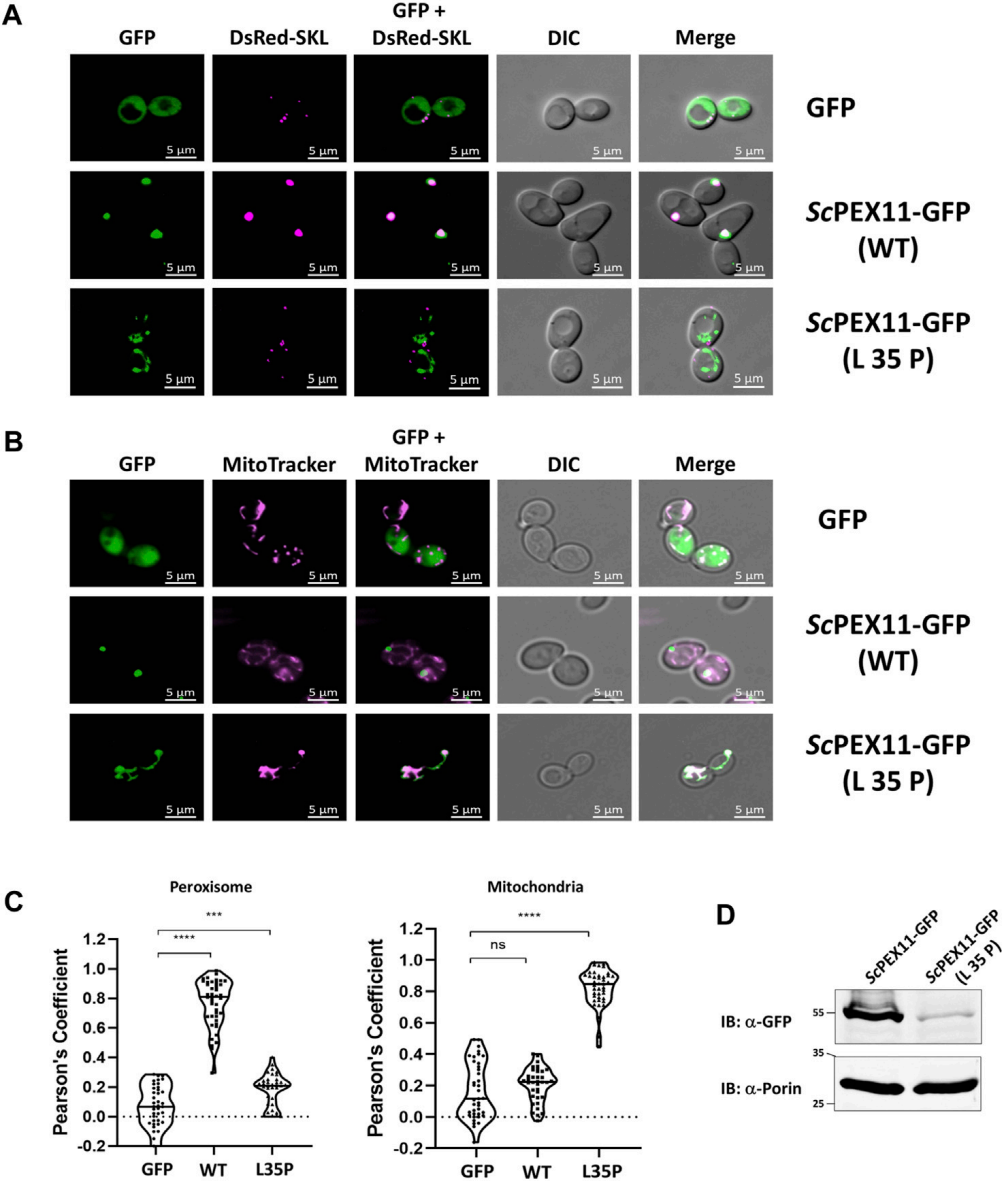
The N-terminal binding site for PEX19 is essential for peroxisomal localization of *Sc*PEX11. Plasmids expressing *Sc*PEX11-GFP (wildtype and L35P-mutant) or the peroxisomal marker protein DsRed-SKL were cotransformed in the BY4742 yeast strain. **(A)** Clones expressing GFP fusion proteins and the peroxisomal marker DsRed-SKL were grown on plates and visualized by fluorescence microscopy. Merged images reveal peroxisomal colocalization of *Sc*PEX11-GFP (wildtype) with DsRed-SKL (pseudo-colored to magenta), indicative for its peroxisomal localization. In contrast, the L35P exchange that is known to block PEX19 binding site result in mislocalization of *Sc*PEX11. **(B)** *Sc*PEX11-GFP with mutation L35P mislocalizes to mitochondria as demonstrated by its colocalization with MitoTracker (pseudo-colored to magenta). DIC–Differential Interference Contrast, Scale bar—5 μm. **(C)** Quantification of the colocalization of *Sc*PEX11-GFP to peroxisomes (left) or mitochondria (right). The Pearson’s coefficient of colocalization to respective organelle is shown. Dots within the violin plot indicates individual Pearson correlation coefficient data points and the central line represents the median. Statistical significances were calculated by one-way ANOVA, with Dunnett’s multiple comparisons test (n ≥ 35 cells). ****, *p* < 0.0001; ***, *p* = 0.0003; ns, not significant. **(D)** Expression of *Sc*PEX11-GFP (wildtype and L35P mutant) was tested by immunoblotting with anti-GFP antibody, which revealed that the steady-state concentration of the L35P is much lower than that of the corresponding wild-type protein. Porin served as the loading control.

In yeast, *Sc*PEX11 contains only one PEX19 binding site, spanning amino acids 27–41 (Rottensteiner et al., 2004), that is homologous to trypanosomal BS1. Mutational analysis of this binding site indicated that the L35P mutation completely abolished interaction with *Sc*PEX19 (Rottensteiner et al., 2004). Here we introduced this mutation into the full-length sequence of PEX11 fused to GFP and analyzed its subcellular localization in comparison to wild-type PEX11 by fluorescence microscopy (Figure 5A). As expected, the full-length PEX11-GFP is targeted to peroxisomes as indicated by its co-localization with the peroxisomal marker DsRed-SKL (Figure 5A, middle panel). The PEX11-GFP fusion harboring the L35P exchange, however, was partly mislocalized to tubular structures (Figure 5A, lower panel). We further performed staining of yeast cells that express PEX11_L35P_-GFP together with a mitochondrial marker (MitoTracker) (Figure 5B, lower panel), confirming that the L35P mutant is mislocalized to mitochondria. Immunoblot analysis of cells shown in Figure 5A show that GFP-tagged wild-type PEX11 is stable, but the steady-state concentration of L35P mutant protein that cannot bind to PEX19 is very low in comparison (Figure 5D).

The data show that PEX11 from *Trypanosoma* and yeast as well as PEX11β from humans contain a conserved N-terminal region that can bind PEX19. This region, corresponding to BS1 in *Trypanosoma*, is required to maintain the steady-state concentration of PEX11 in all studied species, and at least for yeast, it is shown that it is also required for efficient targeting of PEX11 to peroxisomes.

### 3.3 Cryptic N-terminal targeting signal of trypanosomal PEX11

**FIGURE 6:**
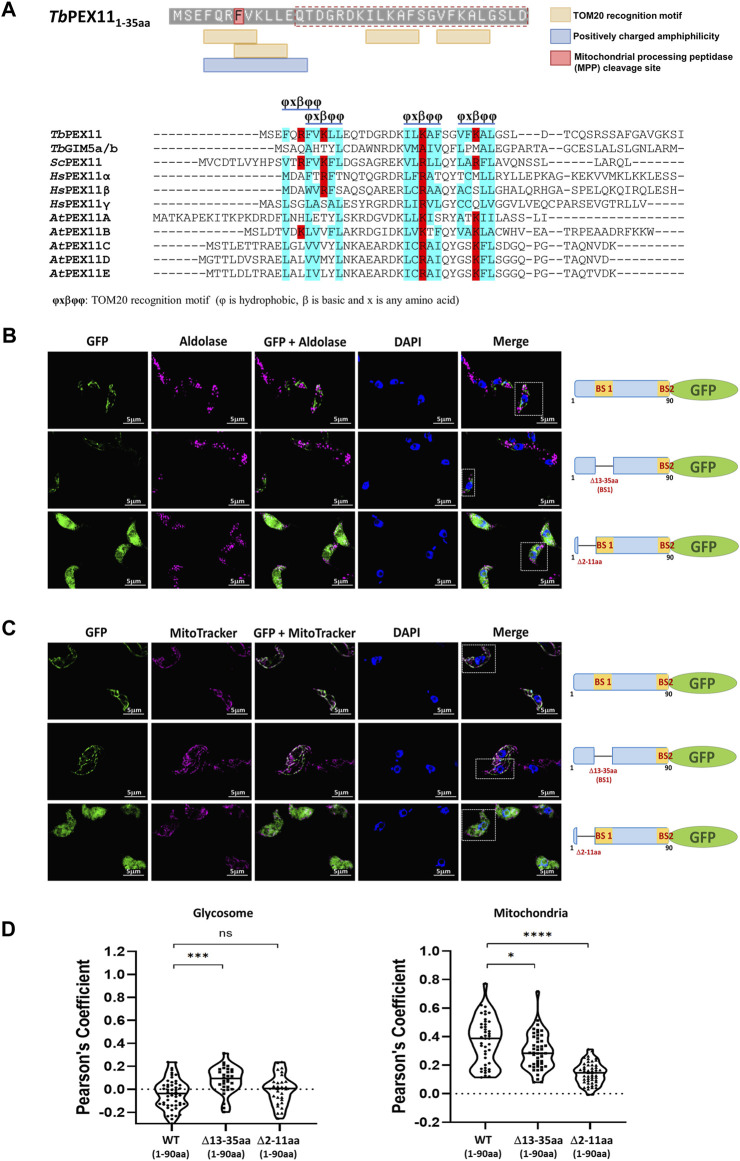
Cryptic signal at the N-terminus causes mitochondrial mislocalization of *Tb*PEX11. **(A)** Upper panel: Prediction of two overlapping putative TOM20 motifs with positively charged amphiphilicity within the N-terminal region *Tb*PEX11 and partially overlapping with the PEX19 binding site region (red dotted box). The residue highlighted in red indicates the presence of a mitochondrial processing peptidase (MPP) cleavage site. The presence of a predicted TOM20 recognition motif and positively charged amphiphilic region with MPP cleavage site were determined by Mitofates webtool (https://mitf.cbrc.pj.aist.go.jp/MitoFates/cgi-bin/top.cgi). Lower panel: Multiple sequence alignment of N-terminal sequences of PEX11 proteins from parasites, yeast, humans, and plants indicates the presence of conserved TOM20 motifs with positively charged (red) residues encased by hydrophobic (blue) amino acid residues. **(B)** Subcellular localization of the N-terminal domain of *Tb*PEX11 (1–90aa) fused to GFP with and without deletion of the PEX19-binding site 1 (Δ13-35aa) or deletion of a N-terminal putative TOM20 binding motif (Δ2-11aa) by fluorescence and immunofluorescence microscopy. The non-truncated fusion with and without PEX19 binding site 1 (BS1) did not co-localize with the glycosomal marker aldolase but was targeted to mitochondrion as shown below (upper and middle panel). The *Tb*PEX11-GFP lacking the TOM20 motif did mislocalize to the cytosol as indicated by the overall cell labelling (lower panel). **(C)** The non-truncated fusion with and without PEX19 binding site 1 (BS1) localized to the mitochondrion, demonstrated by their colocalization with MitoTracker (upper and middle panel, respectively). The glycosomal marker aldolase (pseudo-colored to magenta) was labelled with the corresponding antibody, nuclei and kinetoplasts were stained with DAPI, and mitochondrion was visualized by MitoTracker (pseudo-colored to magenta). Scale bar—5 μm. **(D)** Quantification of the colocalization to glycosomes (left) or the mitochondrion (right). The Pearson’s coefficient of colocalization to respective organelle is shown. Dots within the violin plot indicate individual Pearson correlation coefficient data points and the central line represents the median. Statistical significances were calculated by one-way ANOVA, with Dunnett’s multiple comparisons test (n ≥ 35 cells). ****, *p* < 0.0001; ***, *p* = 0.0001; *, *p* = 0.0262; ns, not significant.

In the absence of PEX19, PMPs are mislocalized to the cytosol and rapidly degraded, or mislocalized to other membranes. For example, PEX3 localizes to the endoplasmic reticulum (Hoepfner et al., 2005) but many PMPs, including yeast PEX11 and PEX13, accumulate in mitochondria when peroxisomes are absent in the cell (Nuebel et al., 2021). PEX13 that is mislocalized to mitochondria can recruit functional docking and import peroxin complexes to mitochondria and also some peroxisomal matrix proteins (Nuebel et al., 2021). Peroxins also accumulate in mitochondria of Zellweger patient-derived cells leading to mitochondrial dysfunction (Nuebel et al., 2021). This can be rescued by overexpressing mitochondrial quality control ATPase ATAD1. We showed that *Tb*PEX11-GFP lacking BS2 is mislocalized to mitochondrion in trypanosomes (Figure 3B, lower panel). However, glycosomal targeting also requires the presence of a transmembrane segment for correct targeting. Accordingly, the *Tb*PEX11 construct containing the first 90 amino acids fused to GFP that lacks the transmembrane domain (TMD) is also mistargeted to mitochondrion. This construct is used here as a control to investigate the requirement for the targeting of *Tb*PEX11 to mitochondria (Figure 6C, upper panel). Normally, mitochondrial proteins are targeted via N-terminal or internal mitochondrial targeting signals (Backes et al., 2018; Bykov et al., 2022), After mitochondrial import, the N-terminal targeting presequences of proteins are removed by mitochondrial processing peptidase (MPP) to allow the proper folding of the imported protein (Kunova et al., 2022). Here, we applied the Mitofates webtool to predict putative mitochondrial targeting signals in the N-terminal region of *Tb*PEX11 (Fukasawa et al., 2015). Although the tool does not identify the *Tb*PEX11-NTD as a classical mitochondrial presequence, it predicts the presence of two tandem TOM20 recognition motifs and a positively charged amphiphilic region with mitochondrial processing peptidase (MPP) cleavage site (Figure 6A, upper panel). The putative TOM20 recognition motifs are in the N-terminal region of *Tb*PEX11 (4–31 aa), partially overlapping with the identified BS1-binding region for *Tb*PEX19 (13–35aa) (Figure 6A, upper panel). Further, we looked for the TOM20 motifs by performing multiple sequence alignment of N-terminal region of PEX11 across organisms (Figure 6A, lower panel), which also contains PEX19 binding site (BS) of yeast (Rottensteiner et al., 2004) and the identified PEX19-BS in *T. brucei* and human (this study). This alignment indicates the conservation of TOM20 motifs in the N-terminal region of PEX11, pointing to its role in mitochondrial mislocalization. To test the putative signal sequences for functionality, we analyzed the role of this region for mitochondrial and glycosomal targeting by fluorescence microscopy (Figure 6B). To this end, GFP tagged N-terminal 90 amino acid region of *Tb*PEX11, with or without BS1 (Δ13-35aa) was analyzed for co-localization with glycosomal marker aldolase (Figure 6B, upper and middle panel). Both fusion proteins were expressed and not targeted to glycosomes but mislocalized to mitochondrion as evident from co-staining with mitochondrial marker (Figure 6C). The expression of these constructs was confirmed by immunoblotting using α-GFP monoclonal antibody (Supplementary Figure S7). Finally, *Tb*PEX11_NTD(1-90aa)_-GFP lacking amino acid residues 2–11, which were predicted to contain TOM20 motifs and positively charged amphiphilicity was analyzed. Expression of this construct did result in a diffuse cytosolic labelling (Figures 6B,C, lower panels), indicating that deletion of this extreme N-terminal region prevented mitochondrial targeting of the fusion protein.

## 4 Discussion

Here we show that the glycosomal membrane protein *Tb*PEX11 contains two PEX19 binding sites in its N-terminal region, as shown by peptide array analysis, yeast two-hybrid studies, pull-down experiments, and AlphaScreen assays. PEX19 is a peroxisomal membrane protein (PMP) receptor and chaperone that stabilizes its cargo proteins and targets them to peroxisomes (Hettema et al., 2000; Sacksteder et al., 2000; Jones et al., 2004). PEX19 binding sites are distributed across the length of cargo proteins, including the C-terminus in case of tail-anchored (TA) proteins (Halbach et al., 2006). Apart from yeast Pex11p (aa 27–41), PEX19 binding sites close to the N-terminus were also found in yeast Pex3p (aa 28–42) and Pxa1p (aa 33–47) (Rottensteiner et al., 2004). Along this line, the N-terminal 95 residues of Pxa1p have recently been shown to be sufficient for targeting a reporter protein to peroxisomes. Interestingly, truncated Pxa1p lacking residues 1–95 still localized to peroxisomes but its targeting depended on the presence of its interaction partner Pxa2 (Jansen et al., 2023).

Eukaryotic organisms contain multiple proteins belonging to the PEX11 family. In yeast, Pex11p contains a single PEX19-BS near its N-terminus, while in the other *Sc*PEX11-family members, Pex25p and Pex27p, binding of PEX19 occurs far distal from the N-terminus (Rottensteiner et al., 2004). A recent study identified a classical PEX19-BS near the N-terminus of *Pichia pastoris* PEX11 (Zientara-Rytter et al., 2022). However, the study also showed that amphipathic helix 4 (H4) located in the C-terminal region of *Pp*PEX11, functions as a second, PEX19-independent mPTS, which is preserved among PEX11-family proteins (Zientara-Rytter et al., 2022). Thus, unlike most PMPs, PEX11 of *Pichia pastoris* can use two mechanisms of transport to peroxisomes, where only one of them depends on its direct interaction with PEX19, but the other does not. The presence of such a PEX19-independent targeting signal is not confirmed in our studies for PEX11 from *T. brucei* and *S. cerevisiae*, as N-terminal mutations or truncations of PEX19-binding sites BS1 or BS2 but not of the amphiphilic helix 4 did prevent efficient glycosomal or peroxisomal targeting of the proteins.

Our data indicate that the N-terminal binding site (BS1) for PEX19 is conserved among *Tb*PEX11 orthologues but not in PEX11-family member GIM5/B of *Trypanosoma* or *Leishmania* (Saveria et al., 2007). Our results also indicate differences within PEX11 isoforms in humans. All three isoforms, PEX11α, PEX11β, and PEX11γ show sequence similarities to the established PEX19 binding site BS1 in yeast and *T. brucei*, but only *Hs*PEX11β did bind human PEX19 (Figures 4B,C). PEX11β is a key factor in the regulation of peroxisome abundance in mammals (reviewed in (Schrader et al., 2016)). It functions as a membrane-remodeling protein that can deform and elongate the peroxisome membrane prior to fission (Delille et al., 2010; Yoshida et al., 2015). Accordingly, PEX11β is the functional counterpart of yeast and *T. brucei* PEX11 that are targeted to their destination in a PEX19-dependent manner and contribute to the morphogenesis of the peroxisomal membrane, which is required for subsequent fission. Overall, the N-terminal PEX11-binding sites for PEX19 are conserved among species. In this study, this is highlighted by the peptide array analysis of *Tb*PEX11, which revealed that human PEX19 binds to the same regions as trypanosomal PEX19 (Supplementary Figure S3).

In the absence of peroxisomes, many PMPs are unstable and degraded or mistargeted to other organelles such as ER and mitochondria. This is seen in yeast as well as in human cells derived from patients suffering from a Peroxisome Biogenesis Disorder (PBD) (Hettema et al., 2000; Nuebel et al., 2021). In yeast, PEX3 localizes to the ER (Toro et al., 2009), while several peroxins/PMPs including PEX13, PEX14, PEX17 (peroxisomal docking complex) as well as PEX11 and PEX25 accumulated on mitochondria (Nuebel et al., 2021). Here we show that mislocalization to mitochondria is seen for *Tb*PEX11 lacking its second PEX19-BS and *Sc*PEX11 harboring mutation of its sole PEX19-binding site, (Figure 3B; 5B). Mitochondria have been described as emergency landing places for abandoned peroxins, which results in a partial reconstitution of the peroxisomal import machinery and routing of a substantial part of the peroxisomal proteome to mitochondria (Nuebel et al., 2021; Vogtle and Meisinger, 2021). The mislocalization of PMPs, especially peroxins, to mitochondria are supposed to be the cause for mitochondrial dysfunction in PBD patients (Hettema et al., 2000; Nuebel et al., 2021). Therefore, it is of interest to gain insight into why some PMPs mislocalize to this particular organelle. Mitochondrial targeting signals (MTS) have been extensively characterized across different organisms and consensus motifs can be predicted. Bioinformatic prediction indicated the presence of positively charged amphiphilicity in the extreme N-terminal Helix 1 and detected four TOM20 recognition motifs in NTD of *Tb*PEX11, out of which two tandem motifs coincide with the N-terminal PEX19-BS (Figure 6A). There is no obvious structural similarity between PEX19 and TOM20. The TOM20 recognizes motifs in cargo proteins via its TPR (tetratricopeptide repeat) domain, which is not present in PEX19. In our *in vitro* studies, PEX19 can directly interact with the PEX19-BSs, without the requirement of an additional cofactor. Surprisingly, the iMTS-Ls predictor also recognizes PEX19-BSs in *Tb* and *Sc*PEX11 as having Internal Matrix Targeting Signal-like Sequences (iMTSL) propensity (Supplementary Figure S8) (Schneider et al., 2021). How these signals that have primary sequence and potential structural similarities, are faithfully recognized by the correct receptor, and targeted to the correct location requires further investigation. The expression of *Tb*PEX11-NTD lacking N-terminal Helix 1 did not any more mistarget to the mitochondrion. This demonstrates that the N-terminal amphipathic helix at the extreme N-terminus of *Tb*PEX11 is essential for the mitochondrial mislocalization. This result indicates that *Tb*PEX11 harbors a cryptic mitochondrial targeting signal. Whether this is also true for human PEX11 and other PMPs, and involvement of TOM complex machinery requires further investigation.

### 4.1 What could be the role of a mitochondrial targeting signal of PEX11?

In mature glycosomes/peroxisomes of the wild type cells, *Tb*PEX11-NTD is exposed to the cytosol. In this case, the cryptic N-terminal signals may be masked by the oligomerization of PEX11. However, in newly formed glycosomes, which are importing PEX11, these signals may be still accessible to interact with the mitochondrial TOM machinery, and this may mediate glycosome-mitochondrion membrane contact site (MCS). Accordingly, association of *Sc*PEX11 with the mitochondrial TOM complex has been seen in two studies, i) 37-fold enrichment of *Sc*PEX11 in the SILAC based interactome of yeast TOM22 (Opalinski et al., 2018), and ii) interaction of *Sc*PEX11 with TOM22 in split-ubiquitin assay (Eckert and Johnsson, 2003). Recently, a nuclear membrane protein Cnm1 (Contact nucleus mitochondria 1) was shown to interact with TOM70, a component of the mitochondrial TOM (translocase of outer membrane) complex (Eisenberg-Bord et al., 2021). This interaction establishes nuclear-mitochondrial contact sites, which are regulated by phosphatidylcholine metabolism. Interestingly, Cnm1 harbors two predicted transmembrane domains close to the N-terminus, while C-terminal end contains internal mitochondrial targeting signal-like (iMTS-L) sequences, which are known to directly bind to TOM70 (Backes et al., 2018). Similarly, PEX11 localized to the glycosomal membrane could still associate with the mitochondrial preprotein import machinery to establish glycosome-mitochondrial contact. Interestingly, PEX11 of parasite *Entamoeba histolytica* shows dual localization to peroxisomes and mitosomes (Verner et al., 2021). In baker’s yeast, PEX11 interacts with Mdm34, a component of the ER–mitochondria encounter structure (ERMES), and act as a peroxisome–mitochondria tether (Mattiazzi Usaj et al., 2015). It has been shown that a mutant form of Mdm34, a component of the ERMES, which impairs ERMES formation and diminishes its association with the peroxisomal membrane protein PEX11, also causes defects in pexophagy (Liu et al., 2018). Along this line, a role for ERMES complex proteins on regulating peroxisome abundance has been reported (Esposito et al., 2019).

We do not yet know whether the newly identified cryptic mitochondrial targeting signal of *Tb*PEX11 is of functional relevance. However, peroxisomes are not only metabolically linked to mitochondria but also share components of their division machinery (Schrader et al., 2015). These include the tail-anchored adaptor proteins FIS1 and MFF, which are dually targeted to both peroxisomes and mitochondria, where they recruit the fission GTPase DRP1 (also known as DNML1) to the organelle membrane (Schrader et al., 2022). In this context, it is interesting to note that targeting of PEX11β to mitochondria induces mitochondrial division in human cells. Accordingly, like PEX11β also *Tb*PEX11 might have the potential to modulate mitochondrial dynamics.

## Data Availability

The original contributions presented in the study are included in the article/Supplementary Material, further inquiries can be directed to the corresponding authors.

## References

[B1] Abe I. Fujiki Y. (1998a). cDNA cloning and characterization of a constitutively expressed isoform of the human peroxin Pex11p. Biochem. Biophys. Res. Commun. 252 (2), 529–533. 10.1006/bbrc.1998.9684 9826565

[B2] Abe I. Okumoto K. Tamura S. Fujiki Y. (1998b). Clofibrate-inducible, 28-kDa peroxisomal integral membrane protein is encoded by PEX11. FEBS Lett. 431 (3), 468–472. 10.1016/s0014-5793(98)00815-1 9714566

[B3] Agrawal G. Subramani S. (2016). De novo peroxisome biogenesis: evolving concepts and conundrums. Biochim. Biophys. Acta 1863 (5), 892–901. 10.1016/j.bbamcr.2015.09.014 26381541 PMC4791208

[B94] Albertini M. Rehling P. Erdmann R. Girzalsky W. Kiel J. A. Veenhuis M. (1997). Pex14p, a peroxisomal membrane protein binding both receptors of the two PTS-dependent import pathways. Cell 89 (1), 83–92. 10.1016/s0092-8674(00)80185-3 9094717

[B4] Anton M. Passreiter M. Lay D. Thai T. P. Gorgas K. Just W. W. (2000). ARF- and coatomer-mediated peroxisomal vesiculation. Cell Biochem. Biophys. 32 (1), 27–36. 10.1385/cbb:32:1-3:27 11330057

[B5] Breidenbach R. W. Beevers H. (1967). Association of the glyoxylate cycle enzymes in a novel subcellular particle from castor bean endosperm. Biochem. Biophys. Res. Commun. 27 (4), 462–469. 10.1016/s0006-291x(67)80007-x 6052476

[B6] Backes S. Hess S. Boos F. Woellhaf M. W. Godel S. Jung M. (2018). Tom70 enhances mitochondrial preprotein import efficiency by binding to internal targeting sequences. J. Cell Biol. 217 (4), 1369–1382. 10.1083/jcb.201708044 29382700 PMC5881500

[B7] Baerends R. J. Faber K. N. Kiel J. A. van der Klei I. J. Harder W. Veenhuis M. (2000). Sorting and function of peroxisomal membrane proteins. FEMS Microbiol. Rev. 24 (3), 291–301. 10.1111/j.1574-6976.2000.tb00543.x 10841974

[B8] Banerjee S. K. Kessler P. S. Saveria T. Parsons M. (2005). Identification of trypanosomatid PEX19: functional characterization reveals impact on cell growth and glycosome size and number. Mol. Biochem. Parasitol. 142 (1), 47–55. 10.1016/j.molbiopara.2005.03.008 15907560

[B9] Bonekamp N. A. Grille S. Cardoso M. J. Almeida M. Aroso M. Gomes S. (2013). Self-interaction of human Pex11pβ during peroxisomal growth and division. PLoS One 8 (1), e53424. 10.1371/journal.pone.0053424 23308220 PMC3538539

[B10] Boutouja F. Stiehm C. M. Mastalski T. Brinkmeier R. Reidick C. El Magraoui F. (2019). Vps10-mediated targeting of Pep4 determines the activity of the vacuole in a substrate-dependent manner. Sci. Rep. 9 (1), 10557. 10.1038/s41598-019-47184-7 31332264 PMC6646403

[B11] Brun R. Schonenberger (1979). Cultivation and *in vitro* cloning or procyclic culture forms of Trypanosoma brucei in a semi-defined medium. Short communication. Acta Trop. 36 (3), 289–292. 43092

[B12] Bykov Y. S. Flohr T. Boos F. Zung N. Herrmann J. M. Schuldiner M. (2022). Widespread use of unconventional targeting signals in mitochondrial ribosome proteins. EMBO J. 41 (1), e109519. 10.15252/embj.2021109519 34786732 PMC8724765

[B13] De Duve C. Baudhuin P. (1966). Peroxisomes (microbodies and related particles). Physiol. Rev. 46 (2), 323–357. 10.1152/physrev.1966.46.2.323 5325972

[B14] Delille H. K. Agricola B. Guimaraes S. C. Borta H. Luers G. H. Fransen M. (2010). Pex11pbeta-mediated growth and division of mammalian peroxisomes follows a maturation pathway. J. Cell Sci. 123 (16), 2750–2762. 10.1242/jcs.062109 20647371

[B15] Eckert J. H. Johnsson N. (2003). Pex10p links the ubiquitin conjugating enzyme Pex4p to the protein import machinery of the peroxisome. J. Cell Sci. 116 (17), 3623–3634. 10.1242/jcs.00678 12876220

[B16] Eisenberg-Bord M. Zung N. Collado J. Drwesh L. Fenech E. J. Fadel A. (2021). Cnm1 mediates nucleus-mitochondria contact site formation in response to phospholipid levels. J. Cell Biol. 220 (11), e202104100. 10.1083/jcb.202104100 34694322 PMC8548916

[B17] Erdmann R. Blobel G. (1995). Giant peroxisomes in oleic acid-induced *Saccharomyces cerevisiae* lacking the peroxisomal membrane protein Pmp27p. J. Cell Biol. 128 (4), 509–523. 10.1083/jcb.128.4.509 7860627 PMC2199900

[B18] Erdmann R. Schliebs W. (2005). Peroxisomal matrix protein import: the transient pore model. Nat. Rev. Mol. Cell Biol. 6 (9), 738–742. 10.1038/nrm1710 16103872

[B19] Esposito M. Hermann-Le Denmat S. Delahodde A. (2019). Contribution of ERMES subunits to mature peroxisome abundance. PLoS One 14 (3), e0214287. 10.1371/journal.pone.0214287 30908556 PMC6433259

[B20] Faber K. N. Keizer-Gunnink I. Pluim D. Harder W. Ab G. Veenhuis M. (1995). The N-terminus of amine oxidase of Hansenula polymorpha contains a peroxisomal targeting signal. FEBS Lett. 357 (2), 115–120. 10.1016/0014-5793(94)01317-t 7805876

[B21] Fukasawa Y. Tsuji J. Fu S. C. Tomii K. Horton P. Imai K. (2015). MitoFates: improved prediction of mitochondrial targeting sequences and their cleavage sites. Mol. Cell Proteomics 14 (4), 1113–1126. 10.1074/mcp.M114.043083 25670805 PMC4390256

[B22] Galland N. de Walque S. Voncken F. G. Verlinde C. L. Michels P. A. (2010). An internal sequence targets Trypanosoma brucei triosephosphate isomerase to glycosomes. Mol. Biochem. Parasitol. 171 (1), 45–49. 10.1016/j.molbiopara.2010.01.002 20138091

[B23] Gao Y. Skowyra M. L. Feng P. Rapoport T. A. (2022). Protein import into peroxisomes occurs through a nuclear pore-like phase. Science 378 (6625), eadf3971. 10.1126/science.adf3971 36520918 PMC9795577

[B24] Ghaedi K. Tamura S. Okumoto K. Matsuzono Y. Fujiki Y. (2000). The peroxin pex3p initiates membrane assembly in peroxisome biogenesis. Mol. Biol. Cell 11 (6), 2085–2102. 10.1091/mbc.11.6.2085 10848631 PMC14905

[B25] Gietz R. D. Woods R. A. (2002). Transformation of yeast by lithium acetate/single-stranded carrier DNA/polyethylene glycol method. Methods Enzymol. 350, 87–96. 10.1016/s0076-6879(02)50957-5 12073338

[B95] Girzalsky W. Hoffmann L. S. Schemenewitz A. Nolte A. Kunau W. H. Erdmann R. (2006). Pex19p-dependent targeting of Pex17p, a peripheral component of the peroxisomal protein import machinery. J. Biol. Chem. 281 (28), 19417–19425. 10.1074/jbc.M603344200 16679311

[B26] Goldman B. M. Blobel G. (1978). Biogenesis of peroxisomes: intracellular site of synthesis of catalase and uricase. Proc. Natl. Acad. Sci. U. S. A. 75 (10), 5066–5070. 10.1073/pnas.75.10.5066 368807 PMC336264

[B27] Gould S. J. Keller G. A. Hosken N. Wilkinson J. Subramani S. (1989). A conserved tripeptide sorts proteins to peroxisomes. J. Cell Biol. 108 (5), 1657–1664. 10.1083/jcb.108.5.1657 2654139 PMC2115556

[B28] Halbach A. Landgraf C. Lorenzen S. Rosenkranz K. Volkmer-Engert R. Erdmann R. (2006). Targeting of the tail-anchored peroxisomal membrane proteins PEX26 and PEX15 occurs through C-terminal PEX19-binding sites. J. Cell Sci. 119 (12), 2508–2517. 10.1242/jcs.02979 16763195

[B29] Halbach A. Lorenzen S. Landgraf C. Volkmer-Engert R. Erdmann R. Rottensteiner H. (2005). Function of the PEX19-binding site of human adrenoleukodystrophy protein as targeting motif in man and yeast. PMP targeting is evolutionarily conserved. J. Biol. Chem. 280 (22), 21176–21182. 10.1074/jbc.M501750200 15781447

[B30] Hasan S. Platta H. W. Erdmann R. (2013). Import of proteins into the peroxisomal matrix. Front. Physiol. 4, 261. 10.3389/fphys.2013.00261 24069002 PMC3781343

[B31] Hettema E. H. Girzalsky W. van Den Berg M. Erdmann R. Distel B. (2000). *Saccharomyces cerevisiae* pex3p and pex19p are required for proper localization and stability of peroxisomal membrane proteins. EMBO J. 19 (2), 223–233. 10.1093/emboj/19.2.223 10637226 PMC305556

[B32] Hilpert K. Winkler D. F. Hancock R. E. (2007). Peptide arrays on cellulose support: sPOT synthesis, a time and cost efficient method for synthesis of large numbers of peptides in a parallel and addressable fashion. Nat. Protoc. 2 (6), 1333–1349. 10.1038/nprot.2007.160 17545971

[B33] Hoepfner D. Schildknegt D. Braakman I. Philippsen P. Tabak H. F. (2005). Contribution of the endoplasmic reticulum to peroxisome formation. Cell 122 (1), 85–95. 10.1016/j.cell.2005.04.025 16009135

[B34] Islinger M. Li K. W. Seitz J. Volkl A. Luers G. H. (2009). Hitchhiking of Cu/Zn superoxide dismutase to peroxisomes--evidence for a natural piggyback import mechanism in mammals. Traffic 10 (11), 1711–1721. 10.1111/j.1600-0854.2009.00966.x 19686298

[B35] Jansen R. L. M. van den Noort M. Krikken A. M. Bibi C. Bohm A. Schuldiner M. (2023). Novel targeting assay uncovers targeting information within peroxisomal ABC transporter Pxa1. Biochim. Biophys. Acta Mol. Cell Res. 1870 (5), 119471. 10.1016/j.bbamcr.2023.119471 37028652

[B36] Jones J. M. Morrell J. C. Gould S. J. (2004). PEX19 is a predominantly cytosolic chaperone and import receptor for class 1 peroxisomal membrane proteins. J. Cell Biol. 164 (1), 57–67. 10.1083/jcb.200304111 14709540 PMC2171958

[B37] Kalel V. C. Erdmann R. (2018). Unraveling of the structure and function of peroxisomal protein import machineries. Subcell. Biochem. 89, 299–321. 10.1007/978-981-13-2233-4_13 30378029

[B38] Kalel V. C. Schliebs W. Erdmann R. (2015). Identification and functional characterization of Trypanosoma brucei peroxin 16. Biochim. Biophys. Acta 1853 (10), 2326–2337. 10.1016/j.bbamcr.2015.05.024 26025675

[B39] Kall L. Krogh A. Sonnhammer E. L. (2007). Advantages of combined transmembrane topology and signal peptide prediction--the Phobius web server. Nucleic Acids Res. 35, W429–W432. Web Server issue). 10.1093/nar/gkm256 17483518 PMC1933244

[B40] Kamoshita M. Kumar R. Anteghini M. Kunze M. Islinger M. Martins Dos Santos V. (2022). Insights into the peroxisomal protein inventory of zebrafish. Front. Physiol. 13, 822509. 10.3389/fphys.2022.822509 35295584 PMC8919083

[B41] Koch J. Pranjic K. Huber A. Ellinger A. Hartig A. Kragler F. (2010). PEX11 family members are membrane elongation factors that coordinate peroxisome proliferation and maintenance. J. Cell Sci. 123 (19), 3389–3400. 10.1242/jcs.064907 20826455

[B42] Krishna C. K. Franke L. Erdmann R. Kalel V. C. (2023). “Isolation of glycosomes from trypanosoma brucei,” in Peroxisomes: Methods and protocols. Editor Schrader M. (New York, NY: Springer US), 33–45. 10.1007/978-1-0716-3048-8_3 36952176

[B43] Kunova N. Havalova H. Ondrovicova G. Stojkovicova B. Bauer J. A. Bauerova-Hlinkova V. (2022). Mitochondrial processing peptidases-structure, function and the role in human diseases. Int. J. Mol. Sci. 23 (3), 1297. 10.3390/ijms23031297 35163221 PMC8835746

[B44] Kunze M. (2023). Computational evaluation of peroxisomal targeting signals in metazoa. Methods Mol. Biol. 2643, 391–404. 10.1007/978-1-0716-3048-8_28 36952201

[B45] Kuravi K. Nagotu S. Krikken A. M. Sjollema K. Deckers M. Erdmann R. (2006). Dynamin-related proteins Vps1p and Dnm1p control peroxisome abundance in *Saccharomyces cerevisiae* . J. Cell Sci. 119 (19), 3994–4001. 10.1242/jcs.03166 16968746

[B46] Lazarow P. B. Fujiki Y. (1985). Biogenesis of peroxisomes. Annu. Rev. Cell Biol. 1, 489–530. 10.1146/annurev.cb.01.110185.002421 3916321

[B47] Leon S. Goodman J. M. Subramani S. (2006). Uniqueness of the mechanism of protein import into the peroxisome matrix: transport of folded, co-factor-bound and oligomeric proteins by shuttling receptors. Biochim. Biophys. Acta 1763 (12), 1552–1564. 10.1016/j.bbamcr.2006.08.037 17011644

[B48] Li X. Gould S. J. (2002). PEX11 promotes peroxisome division independently of peroxisome metabolism. J. Cell Biol. 156 (4), 643–651. 10.1083/jcb.200112028 11839773 PMC2174077

[B49] Lingard M. J. Trelease R. N. (2006). Five Arabidopsis peroxin 11 homologs individually promote peroxisome elongation, duplication or aggregation. J. Cell Sci. 119 (9), 1961–1972. 10.1242/jcs.02904 16636080

[B50] Liu X. Wen X. Klionsky D. J. (2018). Endoplasmic reticulum–mitochondria contacts are required for pexophagy in *Saccharomyces cerevisiae* . Contact 2, 2158. *Thousand Oaks)* 2. 10.1177/2515256418821584 PMC6408953 30859155

[B51] Lorenz P. Maier A. G. Baumgart E. Erdmann R. Clayton C. (1998). Elongation and clustering of glycosomes in Trypanosoma brucei overexpressing the glycosomal Pex11p. EMBO J. 17 (13), 3542–3555. 10.1093/emboj/17.13.3542 9649425 PMC1170691

[B52] Maier A. Lorenz P. Voncken F. Clayton C. (2001). An essential dimeric membrane protein of trypanosome glycosomes. Mol. Microbiol. 39 (6), 1443–1451. 10.1046/j.1365-2958.2001.02333.x 11260462

[B53] Marshall P. A. Dyer J. M. Quick M. E. Goodman J. M. (1996). Redox-sensitive homodimerization of Pex11p: a proposed mechanism to regulate peroxisomal division. J. Cell Biol. 135 (1), 123–137. 10.1083/jcb.135.1.123 8858168 PMC2121026

[B54] Mattiazzi Usaj M. Brloznik M. Kaferle P. Zitnik M. Wolinski H. Leitner F. (2015). Genome-wide localization study of yeast Pex11 identifies peroxisome-mitochondria interactions through the ERMES complex. J. Mol. Biol. 427 (11), 2072–2087. 10.1016/j.jmb.2015.03.004 25769804 PMC4429955

[B55] Mayerhofer P. U. (2016). Targeting and insertion of peroxisomal membrane proteins: eR trafficking versus direct delivery to peroxisomes. Biochim. Biophys. Acta 1863 (5), 870–880. 10.1016/j.bbamcr.2015.09.021 26392202

[B56] McNew J. A. Goodman J. M. (1994). An oligomeric protein is imported into peroxisomes *in vivo* . J. Cell Biol. 127 (5), 1245–1257. 10.1083/jcb.127.5.1245 7962087 PMC2120261

[B57] Meinecke M. Bartsch P. Wagner R. (2016). Peroxisomal protein import pores. Biochim. Biophys. Acta 1863 (5), 821–827. 10.1016/j.bbamcr.2015.10.013 26497277

[B58] Motley A. M. Hettema E. H. (2007). Yeast peroxisomes multiply by growth and division. J. Cell Biol. 178 (3), 399–410. 10.1083/jcb.200702167 17646399 PMC2064844

[B59] Moyersoen J. Choe J. Fan E. Hol W. G. Michels P. A. (2004). Biogenesis of peroxisomes and glycosomes: trypanosomatid glycosome assembly is a promising new drug target. FEMS Microbiol. Rev. 28 (5), 603–643. 10.1016/j.femsre.2004.06.004 15539076

[B60] Murphy M. A. Phillipson B. A. Baker A. Mullen R. T. (2003). Characterization of the targeting signal of the Arabidopsis 22-kD integral peroxisomal membrane protein. Plant Physiol. 133 (2), 813–828. 10.1104/pp.103.027870 12972647 PMC219055

[B61] Neuhaus A. Kooshapur H. Wolf J. Meyer N. H. Madl T. Saidowsky J. (2014). A novel Pex14 protein-interacting site of human Pex5 is critical for matrix protein import into peroxisomes. J. Biol. Chem. 289 (1), 437–448. 10.1074/jbc.M113.499707 24235149 PMC3879566

[B62] Nuebel E. Morgan J. T. Fogarty S. Winter J. M. Lettlova S. Berg J. A. (2021). The biochemical basis of mitochondrial dysfunction in Zellweger Spectrum Disorder. EMBO Rep. 22 (10), e51991. 10.15252/embr.202051991 34351705 PMC8490991

[B63] Opalinski L. Song J. Priesnitz C. Wenz L. S. Oeljeklaus S. Warscheid B. (2018). Recruitment of cytosolic J-proteins by TOM receptors promotes mitochondrial protein biogenesis. Cell Rep. 25 (8), 2036–2043.e5. 10.1016/j.celrep.2018.10.083 30463002 PMC6280124

[B64] Opperdoes F. R. Borst P. (1977). Localization of nine glycolytic enzymes in a microbody-like organelle in trypanosoma brucei: the glycosome. FEBS Lett. 80 (2), 360–364. 10.1016/0014-5793(77)80476-6 142663

[B65] Orth T. Reumann S. Zhang X. Fan J. Wenzel D. Quan S. (2007). The PEROXIN11 protein family controls peroxisome proliferation in Arabidopsis. Plant Cell 19 (1), 333–350. 10.1105/tpc.106.045831 17220199 PMC1820951

[B66] Platta H. W. Girzalsky W. Erdmann R. (2004). Ubiquitination of the peroxisomal import receptor Pex5p. Biochem. J. 384 (1), 37–45. 10.1042/BJ20040572 15283676 PMC1134086

[B67] Platta H. W. Grunau S. Rosenkranz K. Girzalsky W. Erdmann R. (2005). Functional role of the AAA peroxins in dislocation of the cycling PTS1 receptor back to the cytosol. Nat. Cell Biol. 7 (8), 817–822. 10.1038/ncb1281 16007078

[B68] Reichle R. E. Alexander J. V. (1965). Multiperforate septations, Woronin bodies, and septal plugs in Fusarium. J. Cell Biol. 24 (3), 489–496. 10.1083/jcb.24.3.489 19866647 PMC2106583

[B69] Rhodin J. (1954). “Correlation of ultrastructural organization: and function in normal and experimentally changed proximal convoluted tubule cells of the mouse kidney: an electron microscopic study,” (Sweden: Dept. of Anatomy, Karolinska Institutet). PhD Thesis.

[B70] Rottensteiner H. Kramer A. Lorenzen S. Stein K. Landgraf C. Volkmer-Engert R. (2004). Peroxisomal membrane proteins contain common Pex19p-binding sites that are an integral part of their targeting signals. Mol. Biol. Cell 15 (7), 3406–3417. 10.1091/mbc.e04-03-0188 15133130 PMC452593

[B71] Rottensteiner H. Stein K. Sonnenhol E. Erdmann R. (2003). Conserved function of pex11p and the novel pex25p and pex27p in peroxisome biogenesis. Mol. Biol. Cell 14 (10), 4316–4328. 10.1091/mbc.e03-03-0153 14517338 PMC207022

[B72] Rucktaschel R. Thoms S. Sidorovitch V. Halbach A. Pechlivanis M. Volkmer R. (2009). Farnesylation of pex19p is required for its structural integrity and function in peroxisome biogenesis. J. Biol. Chem. 284 (31), 20885–20896. 10.1074/jbc.M109.016584 19451657 PMC2742854

[B73] Sacksteder K. A. Jones J. M. South S. T. Li X. Liu Y. Gould S. J. (2000). PEX19 binds multiple peroxisomal membrane proteins, is predominantly cytoplasmic, and is required for peroxisome membrane synthesis. J. Cell Biol. 148 (5), 931–944. 10.1083/jcb.148.5.931 10704444 PMC2174547

[B74] Saveria T. Halbach A. Erdmann R. Volkmer-Engert R. Landgraf C. Rottensteiner H. (2007). Conservation of PEX19-binding motifs required for protein targeting to mammalian peroxisomal and trypanosome glycosomal membranes. Eukaryot. Cell 6 (8), 1439–1449. 10.1128/EC.00084-07 17586720 PMC1951143

[B75] Schneider K. Zimmer D. Nielsen H. Herrmann J. M. Muhlhaus T. (2021). iMLP, a predictor for internal matrix targeting-like sequences in mitochondrial proteins. Biol. Chem. 402 (8), 937–943. 10.1515/hsz-2021-0185 34218542

[B76] Schrader M. Costello J. Godinho L. F. Islinger M. (2015). Peroxisome-mitochondria interplay and disease. J. Inherit. Metab. Dis. 38 (4), 681–702. 10.1007/s10545-015-9819-7 25687155

[B77] Schrader M. Costello J. L. Godinho L. F. Azadi A. S. Islinger M. (2016). Proliferation and fission of peroxisomes - an update. Biochim. Biophys. Acta 1863 (5), 971–983. 10.1016/j.bbamcr.2015.09.024 26409486

[B78] Schrader M. Reuber B. E. Morrell J. C. Jimenez-Sanchez G. Obie C. Stroh T. A. (1998). Expression of PEX11beta mediates peroxisome proliferation in the absence of extracellular stimuli. J. Biol. Chem. 273 (45), 29607–29614. 10.1074/jbc.273.45.29607 9792670

[B79] Schrader T. A. Carmichael R. E. Islinger M. Costello J. L. Hacker C. Bonekamp N. A. (2022). PEX11β and FIS1 cooperate in peroxisome division independently of mitochondrial fission factor. J. Cell Sci. 135 (13), jcs259924. 10.1242/jcs.259924 35678336 PMC9377713

[B80] South S. T. Gould S. J. (1999). Peroxisome synthesis in the absence of preexisting peroxisomes. J. Cell Biol. 144 (2), 255–266. 10.1083/jcb.144.2.255 9922452 PMC2132891

[B81] Sulter G. J. Verheyden K. Mannaerts G. Harder W. Veenhuis M. (1993). The *in vitro* permeability of yeast peroxisomal membranes is caused by a 31 kDa integral membrane protein. Yeast 9 (7), 733–742. 10.1002/yea.320090707 8368007

[B82] Swinkels B. W. Gould S. J. Bodnar A. G. Rachubinski R. A. Subramani S. (1991). A novel, cleavable peroxisomal targeting signal at the amino-terminus of the rat 3-ketoacyl-CoA thiolase. EMBO J. 10 (11), 3255–3262. 10.1002/j.1460-2075.1991.tb04889.x 1680677 PMC453050

[B83] Tam Y. Y. Torres-Guzman J. C. Vizeacoumar F. J. Smith J. J. Marelli M. Aitchison J. D. (2003). Pex11-related proteins in peroxisome dynamics: a role for the novel peroxin Pex27p in controlling peroxisome size and number in *Saccharomyces cerevisiae* . Mol. Biol. Cell 14 (10), 4089–4102. 10.1091/mbc.e03-03-0150 14517321 PMC207002

[B84] Thoms S. Gartner J. (2012). First PEX11β patient extends spectrum of peroxisomal biogenesis disorder phenotypes. J. Med. Genet. 49 (5), 314–316. 10.1136/jmedgenet-2012-100899 22581969

[B85] Toro A. A. Araya C. A. Cordova G. J. Arredondo C. A. Cardenas H. G. Moreno R. E. (2009). Pex3p-dependent peroxisomal biogenesis initiates in the endoplasmic reticulum of human fibroblasts. J. Cell Biochem. 107 (6), 1083–1096. 10.1002/jcb.22210 19479899

[B86] Tyler K. M. Matthews K. R. Gull K. (2001). Anisomorphic cell division by African trypanosomes. Protist 152 (4), 367–378. 10.1078/1434-4610-00074 11822664

[B87] Van Ael E. Fransen M. (2006). Targeting signals in peroxisomal membrane proteins. Biochim. Biophys. Acta 1763 (12), 1629–1638. 10.1016/j.bbamcr.2006.08.020 17020786

[B88] van Roermund C. W. Tabak H. F. van Den Berg M. Wanders R. J. Hettema E. H. (2000). Pex11p plays a primary role in medium-chain fatty acid oxidation, a process that affects peroxisome number and size in *Saccharomyces cerevisiae* . J. Cell Biol. 150 (3), 489–498. 10.1083/jcb.150.3.489 10931862 PMC2175187

[B89] Verner Z. Zarsky V. Le T. Narayanasamy R. K. Rada P. Rozbesky D. (2021). Anaerobic peroxisomes in Entamoeba histolytica metabolize myo-inositol. PLoS Pathog. 17 (11), e1010041. 10.1371/journal.ppat.1010041 34780573 PMC8629394

[B90] Vogtle F. N. Meisinger C. (2021). Mitochondria as emergency landing for abandoned peroxins. EMBO Rep. 22 (10), e53790. 10.15252/embr.202153790 34414648 PMC8490976

[B91] Voncken F. van Hellemond J. J. Pfisterer I. Maier A. Hillmer S. Clayton C. (2003). Depletion of GIM5 causes cellular fragility, a decreased glycosome number, and reduced levels of ether-linked phospholipids in trypanosomes. J. Biol. Chem. 278 (37), 35299–35310. 10.1074/jbc.M301811200 12829709

[B92] Yoshida Y. Niwa H. Honsho M. Itoyama A. Fujiki Y. (2015). Pex11mediates peroxisomal proliferation by promoting deformation of the lipid membrane. Biol. Open 4 (6), 710–721. 10.1242/bio.201410801 25910939 PMC4467191

[B93] Zientara-Rytter K. M. Mahalingam S. S. Farre J. C. Carolino K. Subramani S. (2022). Recognition and chaperoning by Pex19, followed by trafficking and membrane insertion of the peroxisome proliferation protein, Pex11. Cells 11 (1), 157. 10.3390/cells11010157 35011719 PMC8750153

