# Supplementary material for: Molecular basis of the glycosomal targeting of PEX11 and its mislocalization to mitochondrion in trypanosomes
Source: Front Cell Dev Biol. 2023 Aug 17;11:1213761. doi: 10.3389/fcell.2023.1213761 (PMC10469627; doi:10.3389/fcell.2023.1213761)
Supplement: Supplementary file 1 [file Image5.PDF]

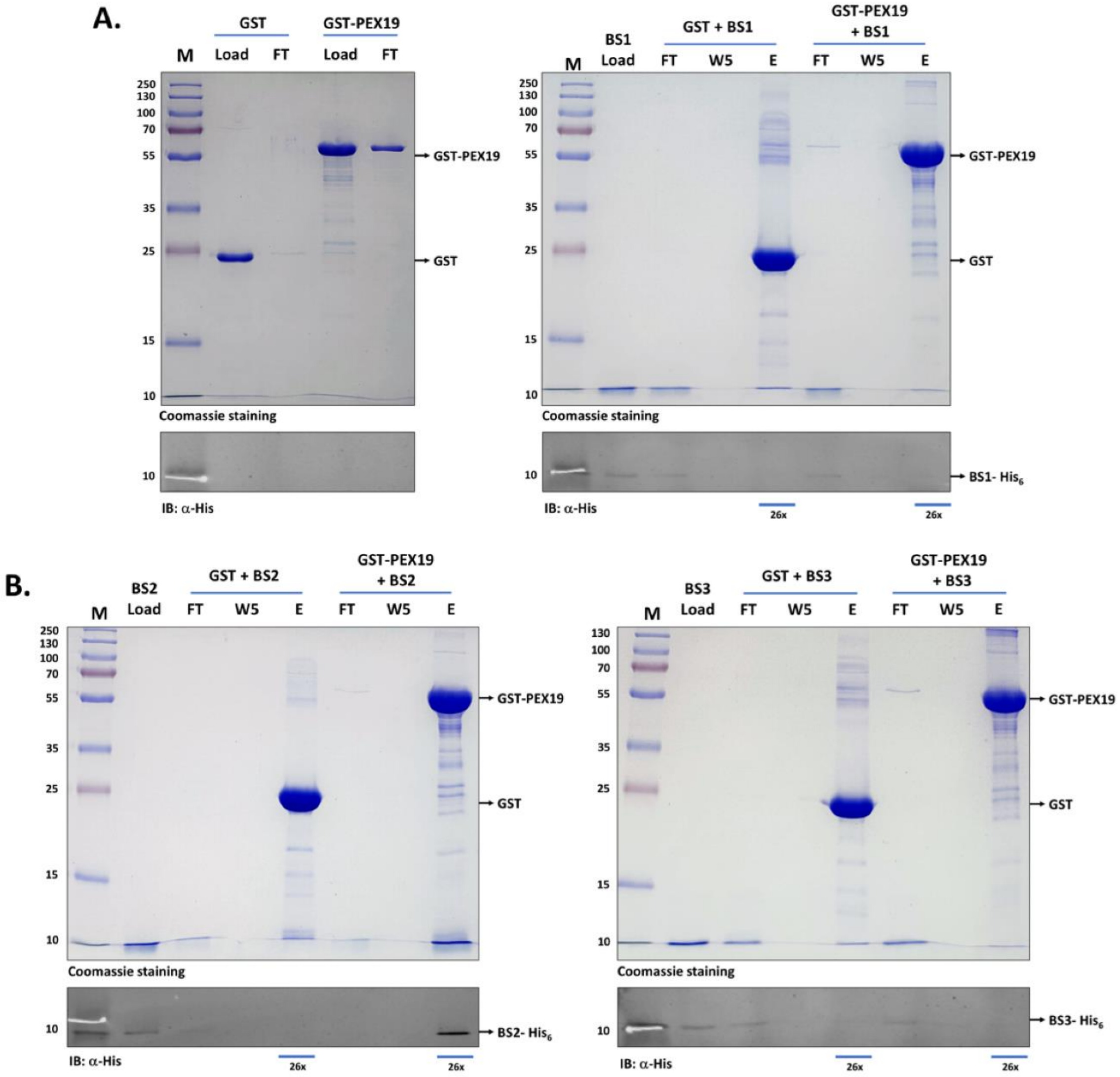

Suppl. Fig. 5. Profiles of GST pull-down assay shown in Fig. 2C. GST (control) and GST-tagged *Tb*PEX19 were immobilized on Glutathione Agarose 4B beads. Subsequently, C-terminally His_6-tagged synthetic peptides, containing the corresponding *Tb*PEX19 binding regions in *Tb*PEX11 (BS1-BS3) were incubated with the immobilized proteins. Following incubation, the proteins bound to the column were eluted and analyzed by SDS-PAGE followed by Coomassie staining and immunoblotting. (**A, left panel**) Profile representing the load and flow through (FT) of the control protein GST and the bait protein GST-tagged PEX19 after incubation with agarose beads. (**A, right panel**) Profile representing the load, FT, washes (W5) and Eluate (E) of the test peptide BS1 after the incubation with bait protein. **(B)** Profiles representing the load, FT, W5 and Eluate of the test peptides BS2 (left panel) and BS3 (right panel) after the incubation with the bait protein. Of the three peptides, only BS2 showed an interaction with GST tagged *Tb*PEX19. The panels below the SDS-PAGEs show the corresponding immunoblot analyses, which were performed using anti-His antibody. Abbreviations: BS, binding site; FT, flow through; W5, wash 5; E, eluate. Eluate fractions were 26X enriched.
