# Supplementary material for: Molecular basis of the glycosomal targeting of PEX11 and its mislocalization to mitochondrion in trypanosomes
Source: Front Cell Dev Biol. 2023 Aug 17;11:1213761. doi: 10.3389/fcell.2023.1213761 (PMC10469627; doi:10.3389/fcell.2023.1213761)
Supplement: Supplementary file 2 [file Image6.PDF]

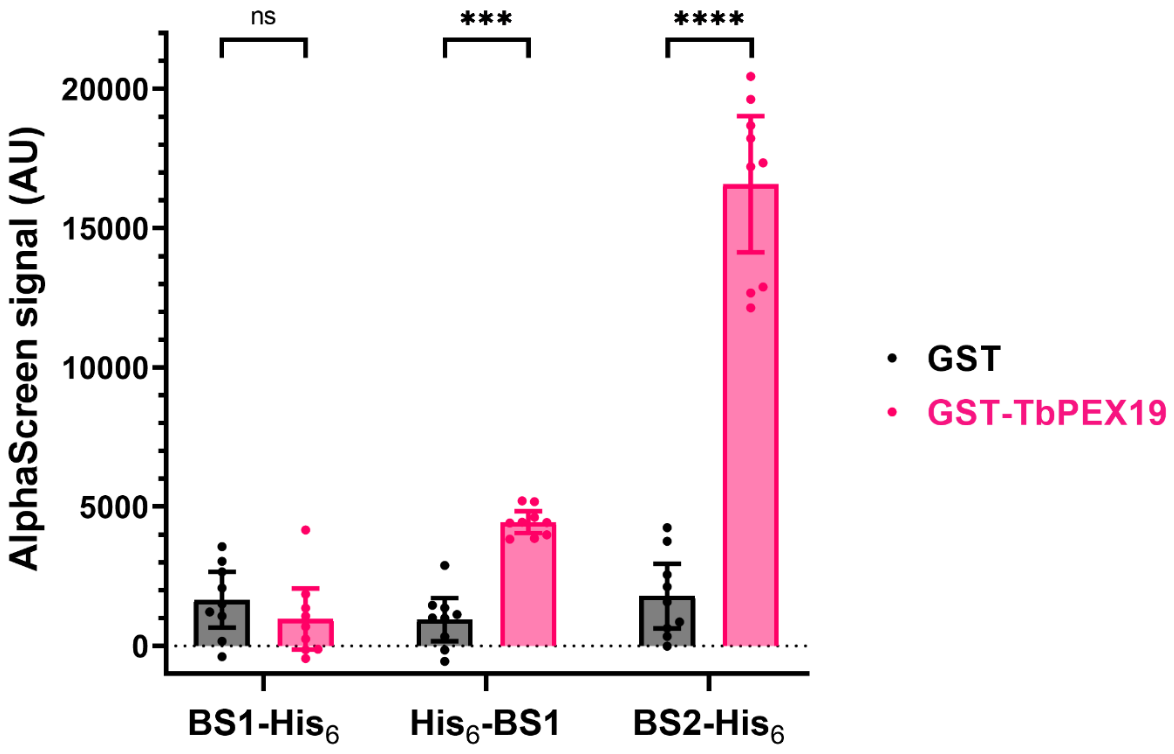

Suppl. Fig. 6. AlphaScreen based assay for *Tb*PEX19-PMP peptide binding. AlphaScreen assay was performed using His$_6$-tagged *Tb*PEX11 binding site peptides (300 nM) with GST-*Tb*PEX19 or GST (30 nM) as negative control. Alpha Signal was monitored using a Synergy Hybrid multimode plate reader. After subtracting the Alpha signal from empty controls (both beads, both beads plus protein/peptide), the values were plotted in GraphPad Prism 9.4.0 using Grouped analysis. Statistical analysis was done using 2way ANOVA with Bonferroni's multiple comparison test. **** $p<0.0001$; *** $p=0.005$; ns, not significant. Error bars represent Mean values with 95% Confidence Intervals of values obtained from three independent biological replicates, each with three technical replicates.
