# Supplementary material for: Molecular basis of the glycosomal targeting of PEX11 and its mislocalization to mitochondrion in trypanosomes
Source: Front Cell Dev Biol. 2023 Aug 17;11:1213761. doi: 10.3389/fcell.2023.1213761 (PMC10469627; doi:10.3389/fcell.2023.1213761)
Supplement: Supplementary file 3 [file Image8.PDF]

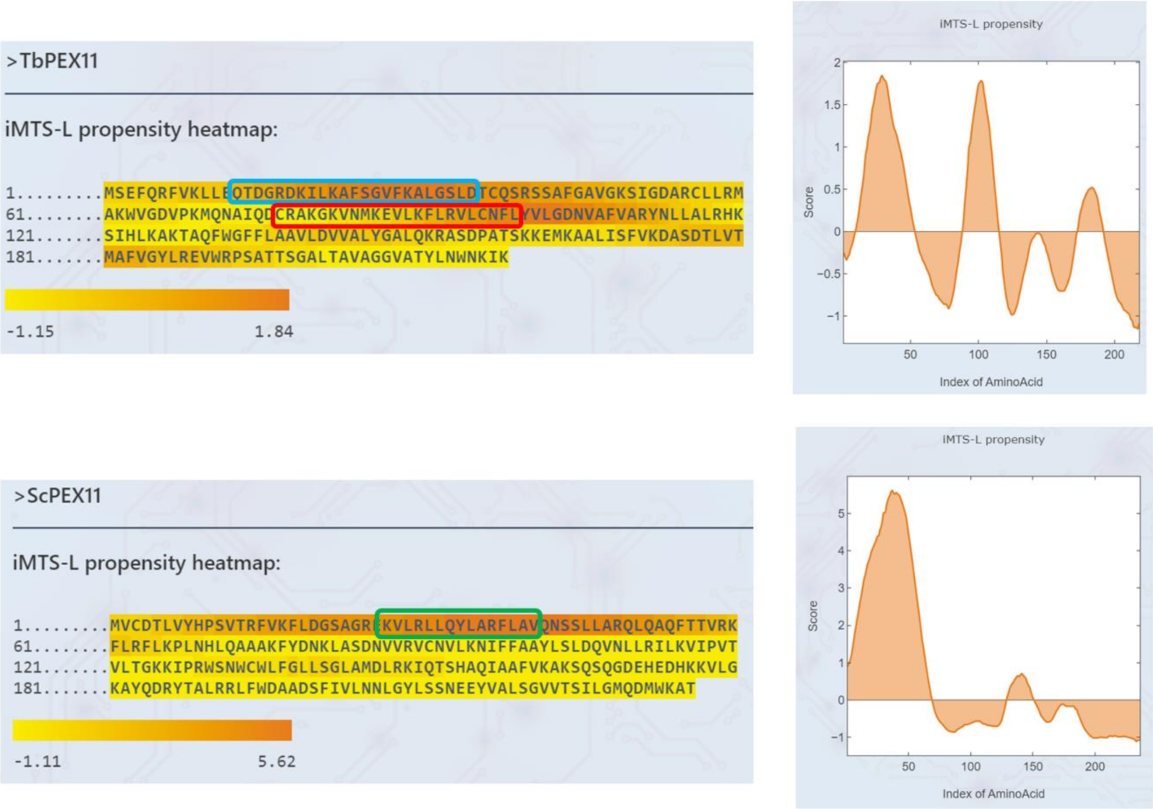

Suppl. Fig. 8. Internal MTS-like signals (iMTS-Ls) prediction reveals an overlap with PEX19 binding sites. The figure shows a screenshot of the prediction of iMTS-L propensity scores with a heatmap. The FASTA sequence of the *Tb*PEX11 and *Sc*PEX11 proteins were used for iMTS-L prediction. The output provides the iMTS-L propensity as a heatmap-colored sequence as well as a probability score profile. The PEX19 binding in the N-terminal region of PEX11 in *S. cerevisiae* (green box) and *T. brucei* (BS1 in blue and BS2 in red box) are predicted with a high probability score by iMTS-Ls. https://csb-imlp.bio.rptu.de/
