# Supplementary material for: Molecular basis of the glycosomal targeting of PEX11 and its mislocalization to mitochondrion in trypanosomes
Source: Front Cell Dev Biol. 2023 Aug 17;11:1213761. doi: 10.3389/fcell.2023.1213761 (PMC10469627; doi:10.3389/fcell.2023.1213761)
Supplement: Supplementary file 4 [file Image4.PDF]

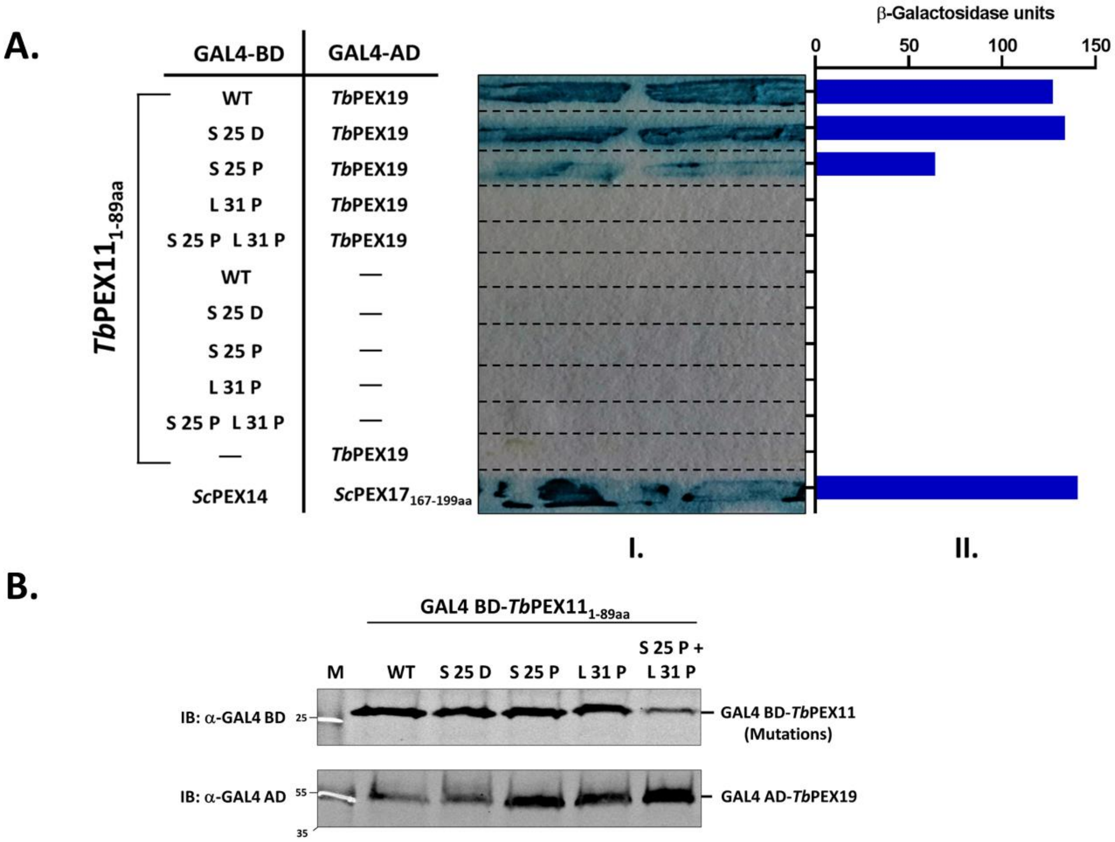

Suppl. Fig. 4. Mutational analysis of the PEX19 binding site 1 (BS1) in *Tb*PEX11. (A) The N-terminal domain of *Tb*PEX11 (NT, 1-89aa), containing the indicated amino acid exchanges, was tested for interaction with full-length *Tb*PEX19 by two-hybrid analysis using colony-lift filter assay (**I**) and liquid ONGP assay (**II**). Colony-lift filter assay were performed with three biological replicates and the β-galactosidase activity units shown are an average of the three technical replicates with one clone. The interaction of *Sc*PEX14-PEX17 served as a positive control. A clear interaction was seen between the wild-type N-terminal domain of *Tb*PEX11 (WT) and *Tb*PEX19. The S to D mutation in PEX11 NT did not influence the PEX19 interaction. However, the S to P mutation significantly reduced the interaction, and the L to P mutation completely abolished the interaction with PEX19. **(B)** Expression of the GAL4-AD / -BD fused PEX19 and PEX11 (WT and mutants) was tested by immunoblotting using monoclonal antibodies against the GAL4-AD and -BD.
