# Supplementary material for: Molecular basis of the glycosomal targeting of PEX11 and its mislocalization to mitochondrion in trypanosomes
Source: Front Cell Dev Biol. 2023 Aug 17;11:1213761. doi: 10.3389/fcell.2023.1213761 (PMC10469627; doi:10.3389/fcell.2023.1213761)
Supplement: Supplementary file 5 [file Image2.PDF]

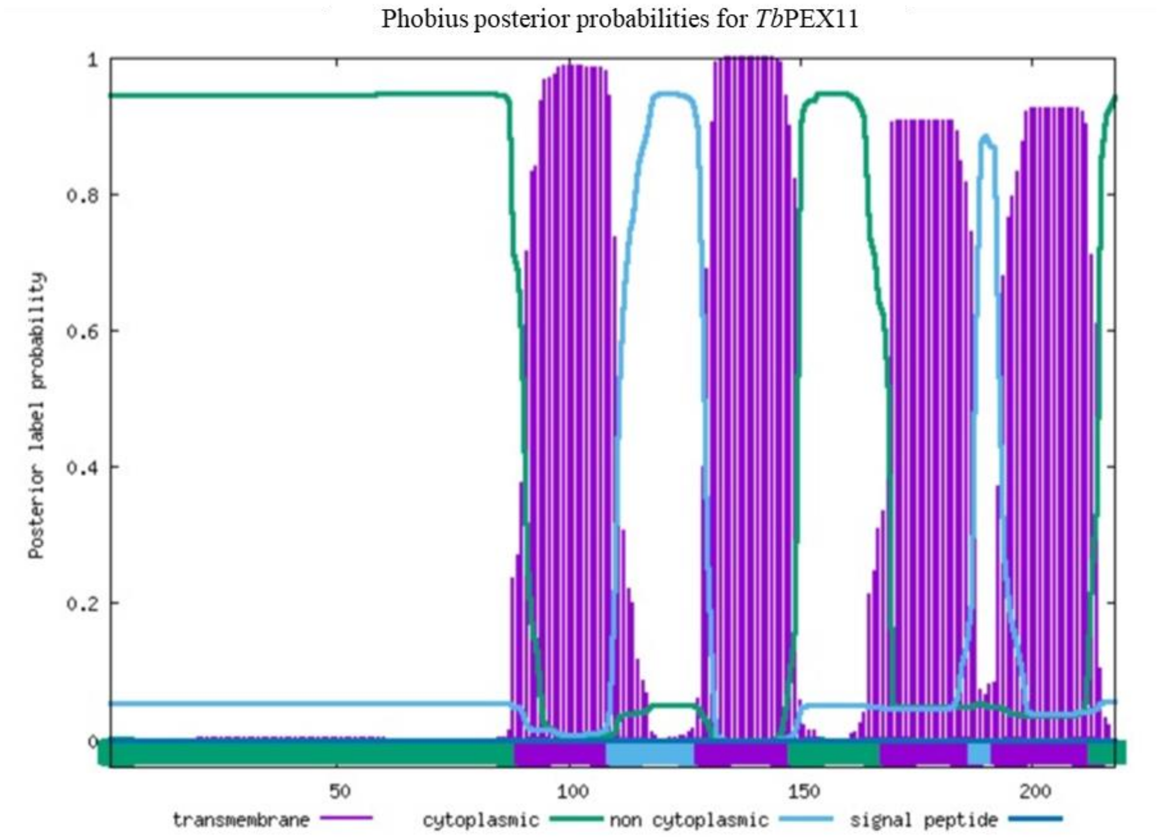

Suppl. Fig. 2. Plot of phobius prediction of transmembrane domains in *Tb*PEX11. The presence of transmembrane segment was determined by the Phobius prediction method (https://phobius.sbc.su.se/). The X-axis shows the predicted results for the region of amino acids whereas the Y-axis stipulates complementary information in the form of probabilities. Magenta color indicates the transmembrane domain, whereas green and blue represents cytosolic and non-cytosolic regions, respectively.
