# Supplementary material for: Molecular basis of the glycosomal targeting of PEX11 and its mislocalization to mitochondrion in trypanosomes
Source: Front Cell Dev Biol. 2023 Aug 17;11:1213761. doi: 10.3389/fcell.2023.1213761 (PMC10469627; doi:10.3389/fcell.2023.1213761)
Supplement: Supplementary file 6 [file Image3.PDF]

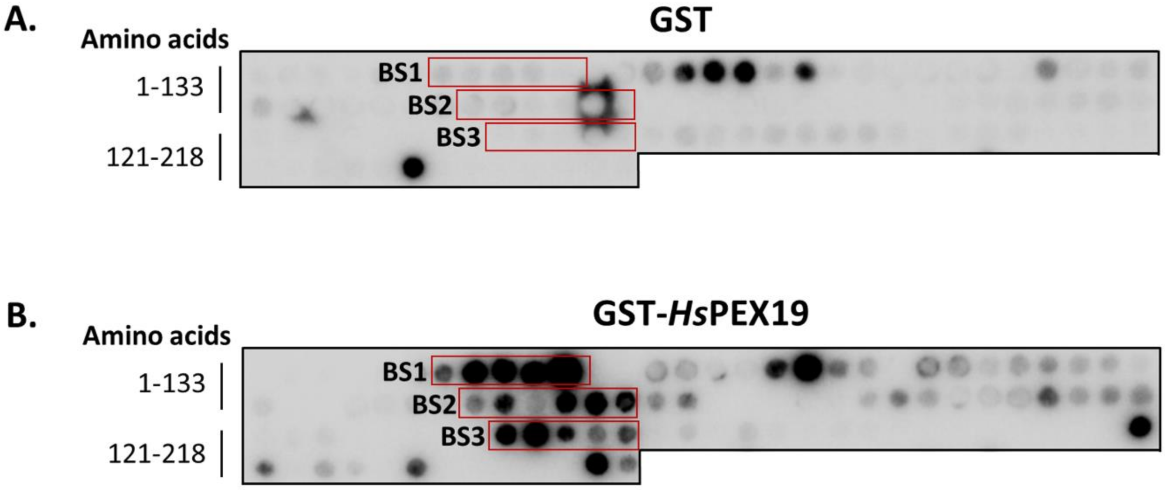

Suppl. Fig. 3. Identification of Human PEX19 binding sites in *Trypanosoma* PEX11. Synthetic 15-mer peptides with 2-amino acids shifts corresponding to the complete *Tb*PEX11 protein sequence were synthesized on cellulose membrane and probed with GST as negative control (**A**) or GST-*Hs*PEX19 (**B**). Bound analyte was immunodetected using primary antibodies against GST and horseradish peroxidase coupled secondary antibody. Signal was detected by chemiluminescence. Three regions in *Tb*PEX11 showed clear interaction with human PEX19 (red boxes, marked BS1-BS3). The three binding regions were the same as the ones detected with *Tb*PEX19 (**Fig. 1**). This result shows that *Tb*PEX11 peptides that bind to *Tb*PEX19 can also interact with human PEX19.
