# Supplementary material for: Molecular basis of the glycosomal targeting of PEX11 and its mislocalization to mitochondrion in trypanosomes
Source: Front Cell Dev Biol. 2023 Aug 17;11:1213761. doi: 10.3389/fcell.2023.1213761 (PMC10469627; doi:10.3389/fcell.2023.1213761)
Supplement: Supplementary file 7 [file Image7.PDF]

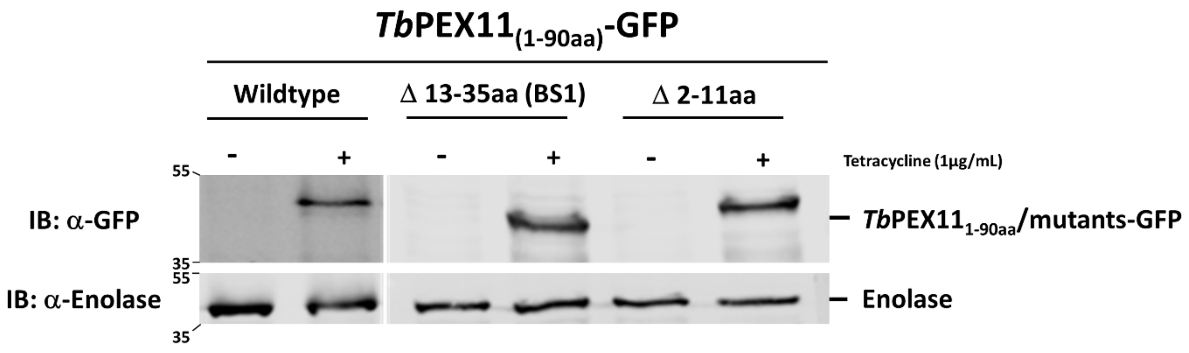

Suppl. Fig. 7. Expression of *Tb*PEX11$_{(1\text{-}90aa)}$-GFP fusion proteins. The expression of the wild-type fusion protein and indicated truncations was tested by immunoblotting using α-GFP monoclonal antibody. Enolase served as the loading control (lower panel).
