# Supplementary material for: Molecular basis of the glycosomal targeting of PEX11 and its mislocalization to mitochondrion in trypanosomes
Source: Front Cell Dev Biol. 2023 Aug 17;11:1213761. doi: 10.3389/fcell.2023.1213761 (PMC10469627; doi:10.3389/fcell.2023.1213761)
Supplement: Supplementary file 8 [file Image1.PDF]

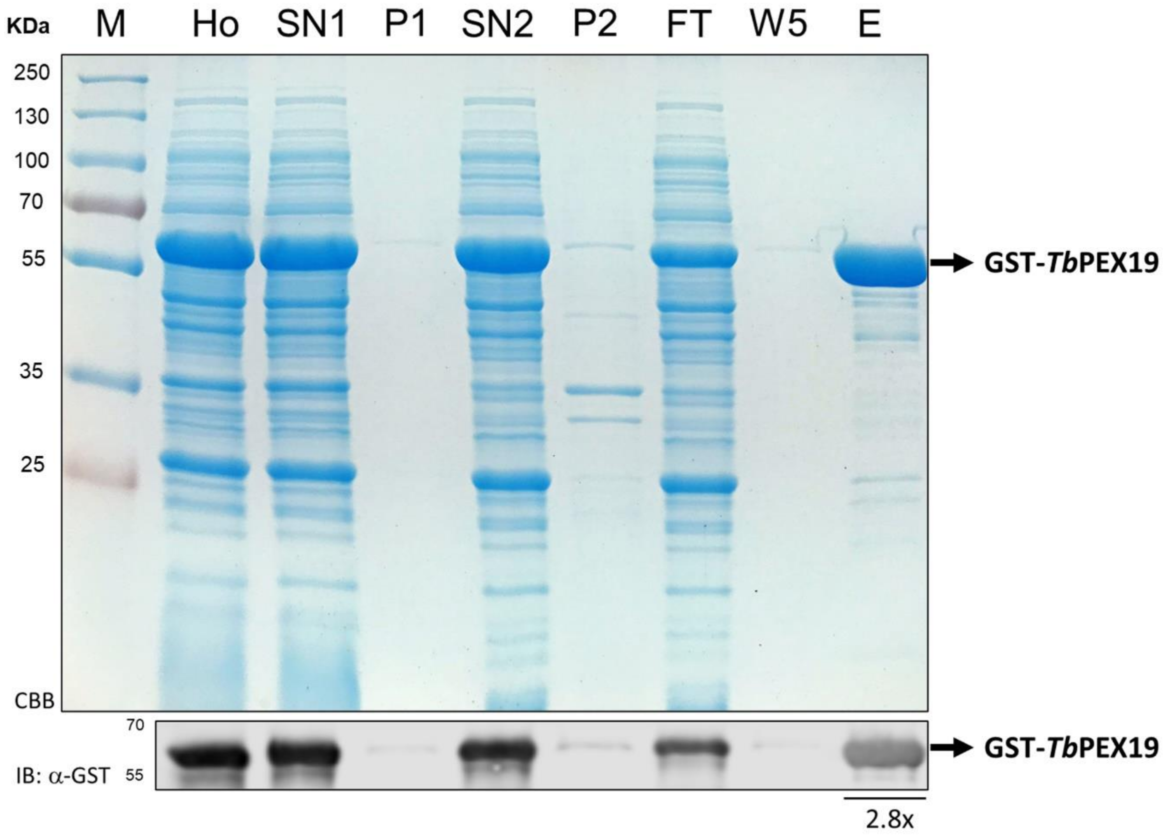

Suppl. Fig. 1. Affinity purification of GST-*Tb*PEX19$_{1\text{-}285aa}$ using Glutathione agarose beads. Cells were grown in LB-medium, and expression was induced with 1mM IPTG (4h at 30 °C). Harvested cells were lysed using the EmulsiFlex-C5 (Homogenate-Ho). Cell debris and unbroken cells (P1) were removed by centrifugation (rotor SX4400, Beckman Coulter, 4,500 rpm for 15 minutes). The resulting supernatant (SN1) was subjected to centrifugation (Rotor SS-34, Thermo Scientific, 14,000 rpm for 1h) to remove non-soluble proteins (P2). The resulting supernatant contained soluble proteins (SN2), which were then loaded onto the Glutathione agarose beads. After collection of the flow-through (FT), the protein-bound beads were washed five times with PBS (W5). GST-tagged proteins were eluted from the beads with 10 mM reduced glutathione (10 mM) in 50 mM Tris-HCl. Samples were taken at each step and analyzed by SDS-PAGE following Coomassie-staining (upper panel) and Immunoblot analysis using the monoclonal Anti-GST antibodies (lower panel).
